# Frequency-potency analysis of IgG+ memory B cells delineates neutralizing antibody responses at single-cell resolution

**DOI:** 10.1016/j.celrep.2024.113948

**Published:** 2024-03-13

**Authors:** Michelle K. Tenggara, Seo-Ho Oh, Catherine Yang, Hardik K. Nariya, Amanda M. Metz, Amit A. Upadhyay, Dedeepya R. Gudipati, Lizheng Guo, Emily G. McGhee, Kiran Gill, Elise G. Viox, Rosemarie D. Mason, Nicole A. Doria-Rose, Kathryn E. Foulds, John R. Mascola, Yuhong Du, Haian Fu, John D. Altman, Qi Yan, Zizhang Sheng, Steven E. Bosinger, Rui Kong

**Affiliations:** 1 Emory Vaccine Center, Atlanta, GA 30329, USA; 2 Emory National Primate Research Center, Atlanta, GA 30329, USA; 3 Vaccine Research Center, National Institutes of Health, Bethesda, MD 20892, USA; 4 Department of Pharmacology and Chemical Biology, Emory University School of Medicine, Atlanta, GA 30322, USA; 5 Emory Chemical Biology Discovery Center, Emory University School of Medicine, Atlanta, GA 30322, USA; 6 Department of Hematology and Medical Oncology and Winship Cancer Institute, Emory University, Atlanta, GA 30322, USA; 7 Department of Microbiology and Immunology, Emory University School of Medicine, Atlanta, GA 30322, USA; 8 Department of Obstetrics and Gynecology, Columbia University Irving Medical Center, New York, NY 10032, USA; 9 Aaron Diamond AIDS Research Center, Columbia University Vagelos College of Physicians and Surgeons, New York, NY 10032, USA; 10 Department of Pathology and Laboratory Medicine, Emory University School of Medicine, Atlanta, GA 30322, USA; 11 Present address: ModeX Therapeutics, Weston, MA 02493, USA; 12 These authors contributed equally; 13 Lead contact

## Abstract

Identifying individual functional B cell receptors (BCRs) is common, but two-dimensional analysis of B cell frequency versus BCR potency would delineate both quantity and quality of antigen-specific memory B cells. We efficiently determine quantitative BCR neutralizing activities using a single-cell-derived antibody supernatant analysis (SCAN) workflow and develop a frequency-potency algorithm to estimate B cell frequencies at various neutralizing activity or binding affinity cutoffs. In an HIV-1 fusion peptide (FP) immunization study, frequency-potency curves elucidate the quantity and quality of FP-specific immunoglobulin G (IgG)+ memory B cells for different animals, time points, and antibody lineages at single-cell resolution. The BCR neutralizing activities are mainly determined by their affinities to soluble envelope trimer. Frequency analysis definitively demonstrates dominant neutralizing antibody lineages. These findings establish SCAN and frequency-potency analyses as promising approaches for general B cell analysis and monoclonal antibody (mAb) discovery. They also provide specific rationales for HIV-1 FP-directed vaccine optimization.

## In brief

Tenggara et al. use a single-cell-derived antibody supernatant analysis (SCAN) workflow to efficiently generate quantitative BCR neutralizing IC50 data against HIV-1, on top of which a two-dimensional frequency-potency analysis delineates quantity and quality of IgG+ memory B cells simultaneously at single-cell resolution.

## Graphical Abstract

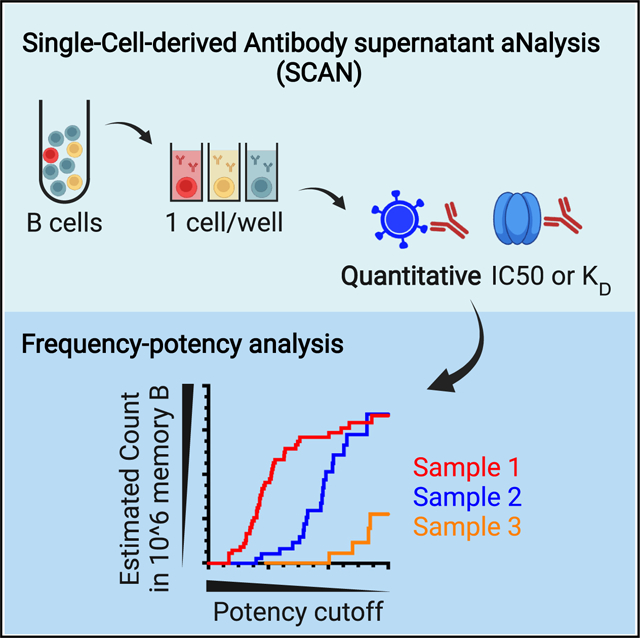

## INTRODUCTION

The overall intensity of a memory B cell response is dominated by the combination of two factors: the frequency of antigen-specific cells^1–3^ and the function of corresponding monoclonal B cell receptors (BCRs) on individual cells.^4–8^ Given the BCR diversity, a two-dimensional profiling of B cell frequency versus monoclonal BCR neutralizing or binding potency (frequency-potency) would be critical to elucidate both quantity and quality of the B cell response to a specific antigen. The frequency of antigen-specific B cells is usually measured by cells with antigen probe staining above a single threshold in flow cytometry.^2,3,9–11^ For example, a tetramer probe is commonly used to stain cells through multivalent binding, although the monovalent BCR affinity required for multivalent binding could be relatively low.^12^ As a result, the frequency of antigen-specific cells accounts for total cells with a broad range of BCR affinity. To determine the frequency-potency of antigen-specific B cells at single-cell resolution, the first step would be generating sufficient quantitative monoclonal BCR functional data from single B cells with minimal selection bias. This step also needs to be labor and cost efficient so it can be practically employed as a routine analysis for multiple samples from multiple studies. These requirements are challenging for commonly used monoclonal antibody (mAb) isolation methods from single B cells.^13^ For instance, multiplex immunoglobulin (Ig) PCR of single antigen-specific B cells cannot eliminate PCR primer bias.^10,14^ Functional screening of whole memory B cells, with^15^ or without^6,16^ microfluidics devices, single-cell RNA sequencing,^17^ or 5′ rapid amplification of cDNA ends (RACE)-based single cell Ig recovery,^18^ may be able to generate quantitative functional data from large amounts of BCRs with minimal bias, but in doing so, most of these methods are still cost and/or labor intensive. Therefore, current approaches still need to be optimized to efficiently generate quantitative mAb functional data.

Vaccine analysis is a field that could benefit from frequency-potency profiling of antigen-specific B cells at single-cell resolution. For example, we previously immunized five non-human primates (NHPs) sequentially with keyhole limpet hemocyanin (KLH)-conjugated HIV-1 fusion peptide (FP) and HIV-1 BG505.DS.SOSIP envelope (Env) soluble trimer.^10^ One animal (DFPH) showed plasma neutralization against multiple heterologous wild-type HIV-1 strains. Using single memory B cell sorting and multiplex Ig PCR approaches, one neutralizing B cell lineage (DFPH-a) was identified from this animal, with the best representative mAb neutralizing 59% of 208 HIV-1 strains in a multiclade panel. The molecular structures and clonal development of this specific lineage were characterized in great detail.^10^ However, due to the lack of high-resolution profiling of the whole FP-specific B cell population for this and other animals in the same group, critical questions remained to be addressed to guide future vaccine optimization. What were the frequency and potency of neutralizing B cells for every animal and time point in this immunization group? Were there similar neutralizing mAbs and lineages in other animals? Was DFPH-a the only dominant neutralizing lineage in animal DFPH? What was the frequency of DFPH-a lineage B cells at different time points? For non-neutralizing and weakly neutralizing antibodies that were previously undercharacterized, were they insufficiently matured, or were they matured to unpreferred directions due to potential distinctions between soluble Env immunogen and native Env trimers on the virus?

To address these general technical needs and specific scientific questions, we developed a comprehensive workflow based on previously reported single B cell culture methods,^16,19–23^ named single-cell-derived antibody supernatant analysis (SCAN), to efficiently generate quantitative BCR functional data prior to Ig sequencing and mAb expression. We also developed an algorithm to estimate the frequency of cells at various BCR neutralization activity (half maximal inhibitory concentration [IC50]) and binding affinity (K_D_) cutoffs and provided two-dimensional frequency-IC50 or frequency-K_D_ curves for antigen-specific IgG+ memory B cells. Using example samples from the HIV-1 FP immunization study described above, the frequency-IC50 curves differentiated animals and B cell lineages to a depth that was not achieved previously. The quantitative neutralizing and binding data on a large number of monoclonal BCRs demonstrated that the binding affinities to soluble HIV-1 Env trimer dominantly determined the neutralizing activities against the virus. With a focus on only neutralizing B cells, a close-to-full recovery rate (96.6%) was achieved, and the DFPH-a lineage was demonstrated to be the only dominantly expanded lineage. Unsurprisingly, top cross-reactive neutralizing mAbs were also efficiently identified after SCAN. Together, these findings established SCAN and the frequency-potency algorithm as promising approaches for general B cell analysis and mAb discovery and provided specific rationales for future HIV-1 FP-directed vaccine optimization.

## RESULTS

### SCAN-derived neutralization titers are accurate and reproducible

**Figure 1.**
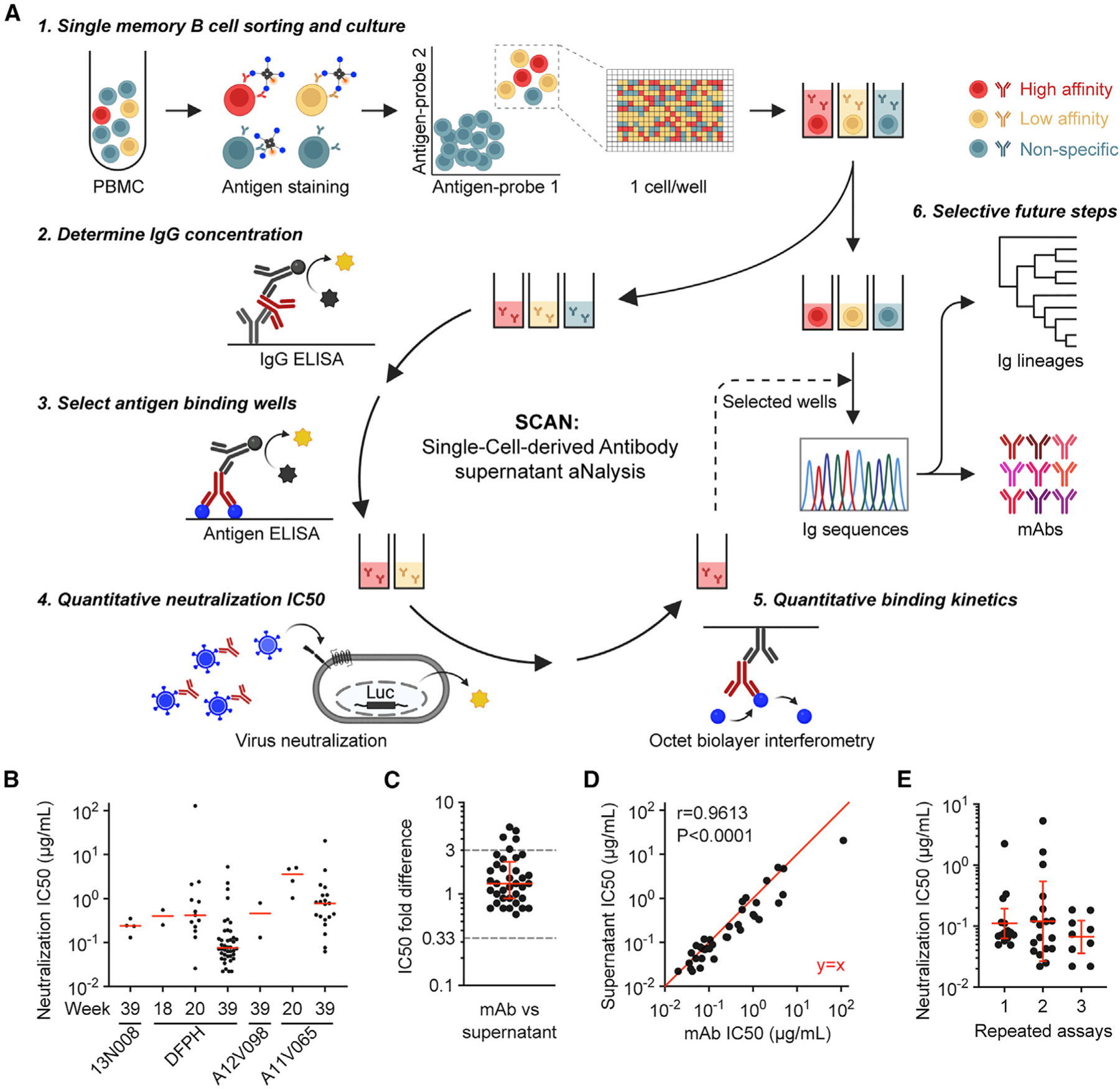
SCAN-derived neutralization titers are accurate and reproducible (A) Schematic of the SCAN workflow. (B) Neutralization IC50 value of single-cell-derived supernatant IgGs. A total of 87 supernatants are shown. Red bars indicate median values. (C) Fold differences in neutralization IC50 values between expressed mAbs (N = 38) and their corresponding supernatants. A red bar and error bars indicate median with interquartile range. (D) Correlation between mAb neutralization IC50 values (N = 38) and their corresponding supernatants. Pearson r and p value are shown. A perfect y = x line is shown in red. (E) Neutralization IC50 values of supernatant IgGs from three repeated SCAN analyses of week 39 PBMCs from animal DFPH. IC50 values were determined for 15, 18, and 9 supernatants in repeated assays 1, 2, and 3, respectively. A red bar and error bars indicate geometric mean and SD. (B–E) All neutralization titers are against HIV-1 Env-pseudotyped virus BG505.N611Q. See also Figures S1 and S2 and Table S1.

To generate quantitative BCR functional data prior to mAb expression, a comprehensive SCAN workflow (Figure 1A) was developed based on single-B cell culture methods.^16,19–23^ Briefly, antigen tetramer probe-stained IgG+ memory B cells were sorted into 384-well plates at a single cell per well and then cultured for 13–21 days before supernatant harvest. The concentration of IgG in the supernatant was determined by sandwich ELISA. All supernatants were also screened in a highly sensitive single-point antigen ELISA. Neutralization activities and binding kinetics were then quantitatively determined for supernatants showing positive antigen binding and with IgG concentration above the threshold. Meanwhile, cells were frozen and used for highly selective Ig sequence recovery, lineage analysis, and mAb assessment.

Here, the main challenge was to generate quantitative neutralizing IC50 data with a small volume of supernatant in which IgG concentration was generally low. We optimized the HIV-1 Env-pseudotyped virus neutralization assay (TZM-bl assay). For a duplicated, multipoint, 5-fold serially diluted assay in 96- and 384-well plates, the previous protocol required 25 and 30 mL of sample, respectively, without accounting for the extra volume needed for the loss during liquid transfer, and the samples were diluted 5 times from the stock at the starting point.^7,24,25^ After optimization, the sample was diluted only 1.67-fold at the starting point with an even lower amount of sample needed (Figure S1A). Thus, while the original protocol was commonly used for qualitative screening of supernatant and detecting highly potent neutralizers, the optimized protocol made it more realistic for quantitative IC50 determination due to increased sensitivity and less sample usage. Given that multiple analyses need to be completed with only a total of 60 mL of supernatant, this improvement also made it possible to generate quantitative IC50 data from many more wells. Additionally, the neutralization curves from the optimized 384-well protocol recapitulated those from the classic 96-well protocol (Figure S1B), demonstrating that the optimized protocol is reliable.

We applied SCAN to 12 peripheral blood mononuclear cell (PBMC) samples collected at weeks 18, 20, and 39 from 4 NHPs that were immunized twice with FP-KLH and three times with HIV-1 BG505.DS.SOSIP Env trimer (Figure S2A). A total of 1821 FP probe-stained IgG+ B cells were sorted. 329 (18% of 1,821) wells showed supernatant IgG greater than 0.15 μg/mL (1 nM). 283 (86% of 329) supernatants showed positive binding (optical density 450 [OD_450_] > 0.1) in a highly sensitive FP ELISA and were assessed for neutralizing activity against HIV-1 BG505.N611Q virus (Figure S2B). Neutralization IC50 values were determined for 87 (31% of 283) supernatants (Figure 1B and S2B; Table S1); for the remaining 196 supernatants, 50% inhibition of virus entry was not reached at the highest tested concentration. Of note, the N611Q variant was selected as it usually increases sensitivity to FP-specific antibody neutralization.^10,14,26^

To examine the accuracy of supernatant neutralization IC50 values, immunoglobulin heavy- and light-chain sequences were recovered from 38 wells representing different animal origins, time points, and supernatant neutralizing IC50 values, and mAbs were produced. mAb neutralizing IC50 values against the same virus were determined using the same neutralization assay protocol. 37 of 38 mAbs (97.4%) showed IC50 values that were similar to those from the corresponding supernatants (Figure 1C). Specifically, 32 (84.2%) showed less than 3-fold IC50 differences between mAbs and corresponding supernatants, and 5 (13.2%) showed 3.1- to 5.4-fold IC50 differences. Of note, 3-fold is the expected variability for this assay.^25^ Overall, the IC50 titers of these 38 mAbs highly correlated with their corresponding supernatants (Pearson r = 0.9613, p < 0.0001) (Figure 1D). Only one well showed a substantial difference (465-fold) between mAb IC50 (73 μg/mL) and supernatant IC50 (0.156 μg/mL), which likely represented an experimental error in any of the steps from supernatant IgG concentration to mAb neutralization. Together these results demonstrated that supernatant neutralization IC50 values accurately reflected the neutralizing potency of the corresponding monoclonal BCRs.

We also tested the reproducibility of SCAN among different experiments. Three vials of week 39 PBMCs from animal DFPH were independently analyzed on different days. The percentages of FP-probe double-positive cells in total IgG+IgM− B cells were highly consistent among three assays (mean ± SD: 0.048% ± 0.005%) (Figures S2C and S2D). Neutralization IC50 values against HIV-1 BG505.N611Q virus were generated from 15, 18, and 9 supernatants with similar geometric means of 0.11, 0.12, and 0.07 μg/mL, respectively (Figure 1E). Overall, the final BCR potency results are highly reproducible, even when the IgG secretion efficiency varies among different vials of PBMCs and repeated assays (Figure S2E).

### BCR staining showed no impact on supernatant IgG concentration in the SCAN workflow

**Figure 2.**
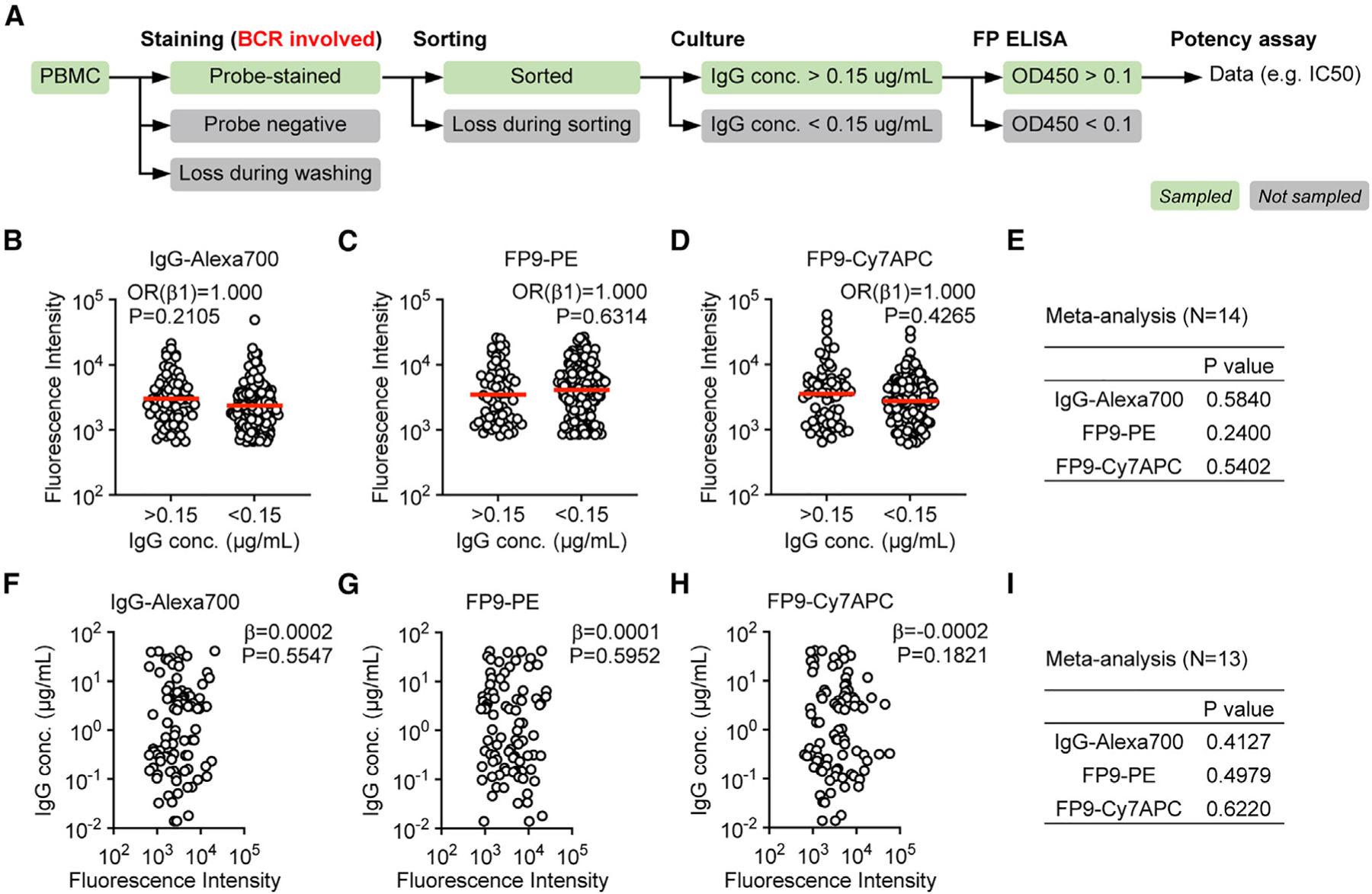
BCR staining showed no impact on supernatant IgG concentration in the SCAN workflow (A) Step-by-step analysis of antigen-specific IgG+ B cell sampling in the SCAN workflow. Green and gray text boxes indicate cells or single-cell culture supernatants. The red text highlights that staining is the only process that involves BCRs and potentially interferes with B cell proliferation and IgG secretion in the culture. (B–D) Fluorescence intensity of IgG-Alexa 700 (B), FP9-PE (C), and FP9-Cy7APC (D) staining in single-cell index sorting. Each open circle indicates a sorted cell. Cells are categorized into two groups with supernatant IgG concentration above or below 0.15 μg/mL after single-cell culture. A red bar indicates geometric mean. Univariable logistics analyses were performed with odds ratio (OR) β1 and p value shown. Data are from a representative assay on week 39 PBMCs from animal A11V065. (E) Meta-analyses of logistics analysis results from 14 datasets. p values are shown. (F–H) IgG concentrations in single-cell culture supernatants. Each open circle indicates a supernatant. Only those with IgG concentration above 0.15 μg/mL are shown. The x axis shows the fluorescence intensity of IgG-Alexa 700 (F), FP9-PE (G), and FP9-Cy7APC (H) staining of the corresponding sorted cell. Univariable linear regression analyses were performed, with slope (b) and p value shown. Data are from the same assay as in (B)–(D). (I) Meta-analyses of linear regression results from 13 datasets. p values are shown. Of note, one of the original 14 datasets contained only 2 supernatants with IgG concentration above 0.15 μg/mL and so was not qualified for linear regression analysis. See also Figure S3.

To profile BCR function for the whole circulating IgG+ memory B cell population in an unbiased fashion, it is important to examine whether obtaining or losing data is related to the BCR function. In the SCAN workflow, IgG+ memory B cells may be screened out or lost in the following situations (Figure 2A): (1) random loss of cells during staining and washing prior to flow cytometry; (2) cells not sorted because of undetectable antigen probe staining; (3) random loss of antigen probe-stained cells during the sorting, which could be minimized by decreasing the speed of cell flow; (4) wells with supernatant IgG concentration below the threshold for functional assays; and (5) wells with supernatant IgG secreted but showing undetectable binding in a highly sensitive antigen ELISA, which are likely false-positive hits from the sorting. Among all of these, cell loss in situations 1 and 3 is random, and cell screening in situations 2 and 5 is designed to remove non- or low-binding BCRs. Only situation 4 is potentially biased toward BCR function. If the bias exists and is a result of the SCAN workflow, then the most likely reason would be BCR clustering due to antibody or probe staining for flow cytometry, which is the only procedure directly involving BCRs in the entire workflow from PBMCs to IgG supernatant collection.

To test whether BCR staining impacts the supernatant IgG concentration, we performed two-step statistical analyses on a total of 14 datasets. First, the logistic analysis showed that, whether supernatant IgG concentration is above or below 0.15 μg/mL was not related to the fluorescence intensity of IgG-Alexa 700 (meta-analysis p = 0.5840), FP9-PE (meta-analysis p = 0.2400), or FP9-Cy7APC staining (meta-analysis p = 0.5402) (Figures 2B–2E and S3A–S3C). Second, the linear regression analysis showed that the IgG concentration was not correlated with the fluorescence intensity of IgG-Alexa700 (meta-analysis p = 0.4127), FP9-PE (meta-analysis p = 0.4979), or FP9-Cy7APC staining (meta-analysis p = 0.6220) when it was above 0.15 μg/mL (Figures 2F–2I and S3D–S3F). These results demonstrated that the data absence due to undetectable or low IgG secretion in situation 4 was unrelated to BCR staining. Therefore, culture of exclusively antigen probe-stained B cells, instead of large numbers of unstained whole-memory B cells, does not lead to a bias in supernatant IgG analysis. Although unknown bias could still exist, the single-cell BCR dataset from antigen-probe-stained B cell culture is likely a close representation of the whole circulating IgG+ memory B cells.

### Frequency-IC50 curves differentiate the quantity and neutralizing activity of antigen-specific IgG+ memory B cells for different animals and time points

**Figure 3.**
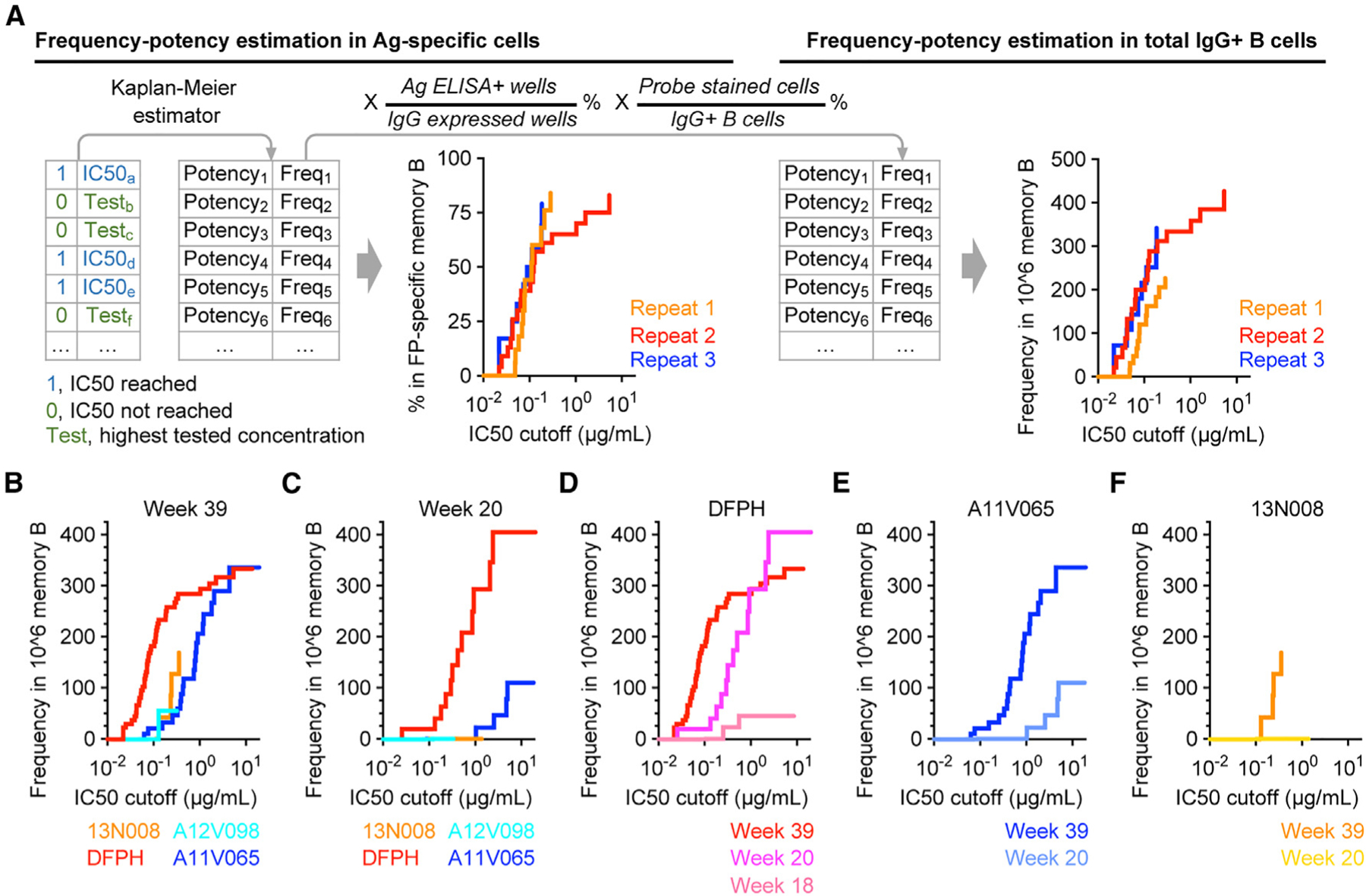
Frequency-potency of neutralizing cells in antigen-specific and total IgG+ memory B cells (A) Frequency-potency estimation in antigen-specific or total IgG+ B cells. The data process is shown step by step, along with example frequency-potency curves. Kaplan-Meier estimator was applied to neutralization IC50 values of all supernatants with IgG concentration above 0.15 μg/mL and FP ELISA OD_450_ above 0.1. The resulting frequency-IC50 curve shows estimated frequencies of antigen-specific IgG+ B cells at the indicated IC50 cutoffs. To estimate the frequencies in total IgG+ B cells, the frequency numbers in the antigen-specific population were further adjusted by the enrichment factors in probe staining and antigen ELISA. Example curves are shown using three repeated assays on the DFPH week 39 sample. (B–F) Frequency-IC50 curves showing the frequency of FP-directed neutralizing cells among total IgG+ B cells. PBMCs from 4 animals are compared at week 39 (B) and week 20 (C). PBMCs from different time points are also compared for individual animals DFPH (D), A11V065 (E), and 13N008 (F).

Using data generated from the example study (Figure S2B), Kaplan-Meier analysis was employed to estimate the frequency of cells at continuous neutralization IC50 cutoffs in FP-specific IgG+ B cells (Figure 3A). Overall, the resulting frequency-IC50 curves were highly consistent among three independently repeated analyses of week 39 DFPH PBMCs, again suggesting that data from SCAN and the frequency-IC50 analysis were reproducible. Of note, according to the Kaplan-Meier algorithm, more data points contributed to the frequency estimation at lower IC50 cutoffs (higher potency, left side of the curve) than to the frequency at higher IC50 cutoffs (lower potency, right side of the curve). Therefore, it is understandable that the left sides of the curves were more consistent than the right sides (Figure 3A).

The frequency-IC50 curve of the antigen-specific population described the relative distribution of BCR neutralizing activity within an individual animal. Considering that the size of the antigen-specific population varies among samples, it is important to use frequency of total IgG+ B cells when comparing different samples. To generate cell frequency in total IgG+ B cells, the frequency of the antigen-specific population was multiplied by two factors: (1) the percentage of probe-stained cells in IgG+ B cells in flow cytometry and (2) the percentage of positive binding wells in IgG expressed wells in the antigen ELISA. After that, the new frequency-IC50 curves graphed the number of cells in one million IgG+ memory B cells against various neutralization IC50 cutoffs (Figure 3A). Notably, the new curves were still similar among three repeat analyses of week 39 DFPH PBMCs.

Several findings were immediately illustrated when SCAN was applied and frequency-IC50 of FP-specific IgG+ B cells were compared among NHP samples from the example study. (1) At week 39, after three Env boost immunizations, FP-specific BCRs that neutralize the BG505.N611Q virus were detectable in all 4 animals (Figure 1B); however, the frequency and potency of top neutralizing B cells in animal DFPH were substantially higher than those in other animals (Figure 3B). Specifically, the neutralization IC50 values of the top 200 neutralizers in one million IgG+ memory B cells in animals DFPH and A11V065 were below about 0.1 and 1 μg/mL, respectively. This explained why the DFPH week 39 plasma neutralization titer was about 10-fold higher than A11V065 plasma (Figure S4A), while the frequency of total FP-binding B cells in animal A11V065 was about 3-fold higher than that in animal DFPH at week 39 (Figure S4B). (2) At week 20, after the first Env boost, the frequency-IC50 of FP-neutralizing B cells in animal DFPH was already substantially higher than in animals A11V065 and 13N008 (Figure 3C). (3) At week 18, prior to Env boost immunization, the frequency of total FP-binding B cells was generally low in all animals (Figure S4B). Interestingly, two neutralizing B cells were identified in animal DFPH (Figure 1B), suggesting that neutralizing activity could be developed by FP immunization only, prior to Env immunogen boost. (4) The frequency-IC50 of FP-specific B cells was clearly improved in individual animals after repeated Env boost immunizations from week 18 to weeks 20 and 39 (Figures 3D–3F). Together, these results delineated both the quantity and quality of FP-directed neutralizing B cells in individual animals at single-cell resolution, which was not achieved in our previous study.^10^

### BCR affinity to soluble HIV-1 Env SOSIP trimer is the main determinant of vaccine-induced FP-directed neutralizing activities

In contrast to the neutralization IC50, which describes the biological function of BCRs against the whole virus by interacting with viral surface antigen, binding affinity measures the physical interaction between BCRs and a soluble antigen, which is usually artificially designed. When a soluble antigen is employed as an immunogen, it is critical that the binding affinity to the antigen correlates with the anticipated neutralizing activity so that the BCR affinity maturation can be driven in a preferred direction. In our example study, the FP-KLH prime and soluble HIV-1 Env trimer boost strategy was designed to induce FP-directed neutralizing antibodies in NHPs. It is still unclear whether non-neutralizing or weakly neutralizing BCRs from the vaccination are mainly due to insufficient affinity maturation against the soluble trimer immunogen or other unknown reasons. For example, a potential mechanism of non-neutralization could be the steric hindrance effect from viral membrane, given that HIV-1 FP is close to the viral membrane,^14^ and the viral membrane is not included in the immunogen.

**Figure 4.**
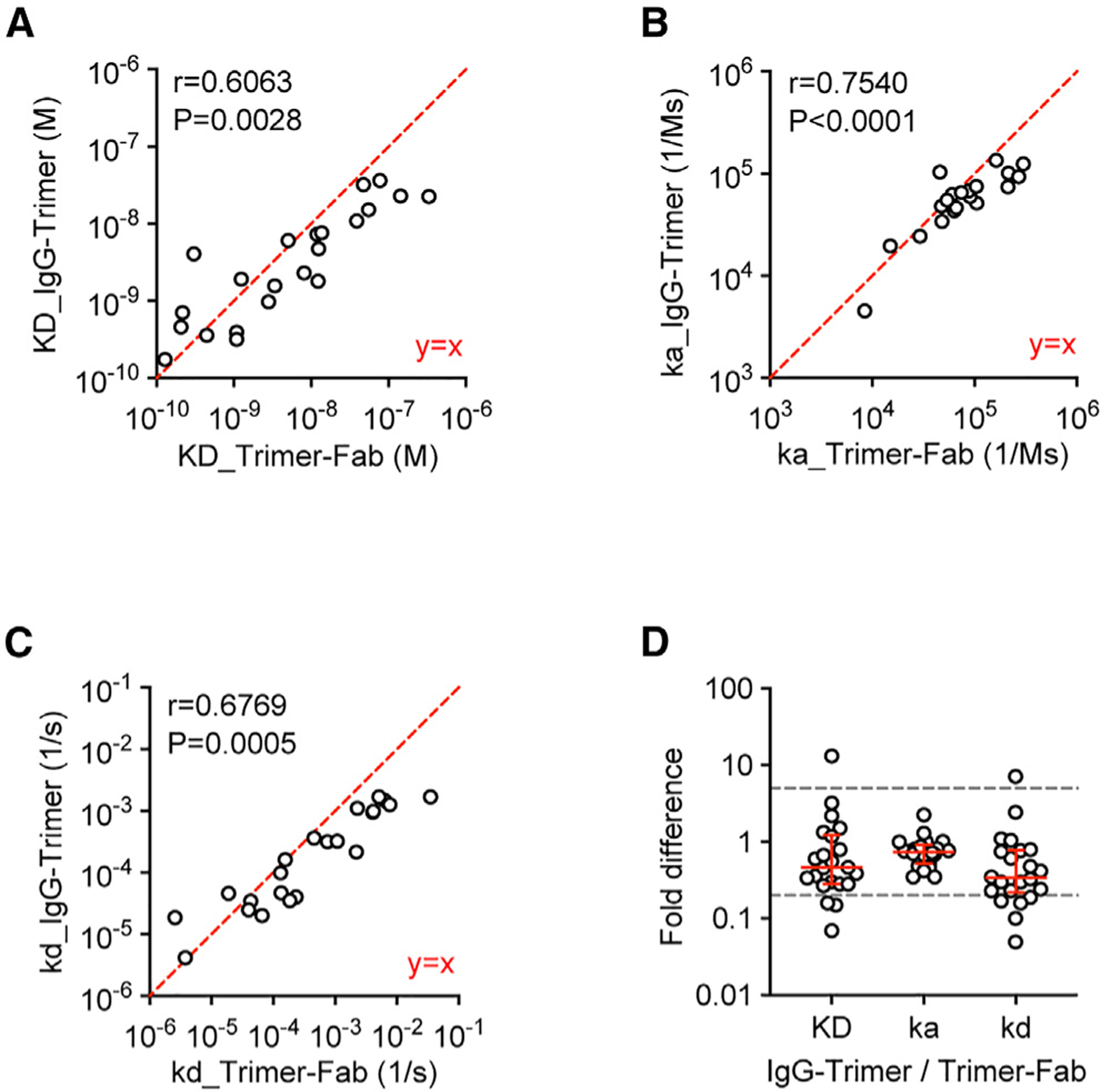
Binding kinetics data on reference mAbs correlate between the single-curve IgG-Trimer method and multi-curve Trimer-Fab method A panel of 22 FP-directed mAbs was tested. The IgG-Trimer method immobilized IgG on the Octet sensor and tested soluble BG505.DS.SOSIP.S613A trimer as an analyte. The Trimer-Fab method immobilized soluble trimer and tested Fab as an analyte. (A–C) Pearson correlation analysis of K_D_ (A), K_a_ (B), and K_d_ (C). Each circle indicates a reference mAb. Pearson r and p values are shown. A perfect y = x line is shown in red. (D) Fold difference of K_D_, K_a_, and K_d_ values. Each circle indicates a reference mAb. A red bar and error bars indicate median and interquartile range. Gray lines indicate 5-fold differences (ratio of 5 or 0.2). See also Figures S5 and Table S2.

To measure the monovalent antibody binding affinity to a soluble antigen, a classic approach usually immobilizes the antigen and measures the binding of free antibody fragments (Fabs) in solution. This method requires mAb isolation followed by Fab production and global fitting of binding curves at multiple Fab concentrations, so it is challenging when analyzing large amounts of B cells. Here, we assessed the apparent binding kinetics directly using single-cell-derived supernatant IgG.^22,27,28^ The supernatant IgG was immobilized on the Octet biosensor, and the soluble Env trimer was applied in solution. A binding curve at a single trimer concentration was fitted to generate apparent binding constants (K_D_, K_a_, and K_d_). Using a panel of 22 FP-specific IgGs that were isolated from a previous study (Figure S5; Table S2), the apparent K_D_, K_a_, and K_d_ generated by this protocol (named IgG-Trimer) significantly correlated with those generated by the classic approach (named Trimer-Fab) (Figures 4A–4C), and the fold differences of K_D_, K_a_, and K_d_ on an individual antibody between the two approaches were mostly within 5-fold (Figure 4D). These results confirmed that the IgG-Trimer protocol can differentiate high to low binding affinities (K_D_) within a range of 0.1–100 nM.

**Figure 5.**
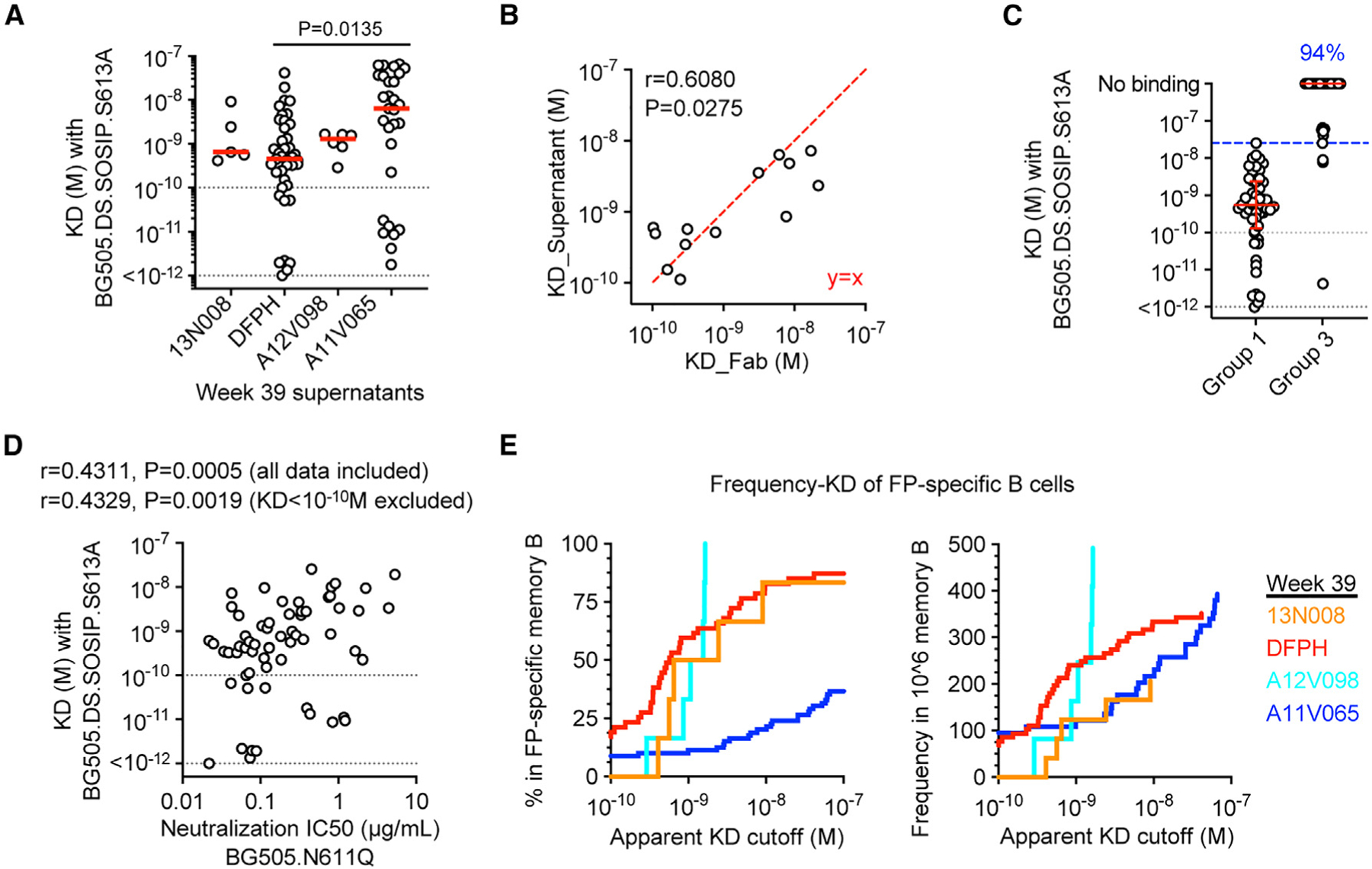
BCR affinity to soluble HIV-1 Env SOSIP trimer is the main determinant of vaccine-induced FP-directed neutralizing activities (A) Apparent K_D_ values of week 39 supernatant IgGs measured by IgG-Trimer method. The BG505.DS.SOSIP.S613A trimer was used. Each circle indicates a supernatant. A red bar indicates median value. Kruskal-Wallis test (p = 0.0249) and Dunn’s multiple-comparisons test were performed. p values from significant pairs are shown. (B) Pearson correlation analysis of K_D_ generated directly from supernatant IgGs or from corresponding Fabs. Each circle indicates a supernatant. Pearson r and p values are shown. A perfect y = x line is shown in red. (C) Apparent K_D_ values of group 1 and group 3 supernatants with the BG505.DS.SOSIP.S613A trimer. Group 1 includes supernatants with BG505.N611Q neutralization IC50 values below 1 μg/mL. Group 3 includes non-neutralizing supernatants or supernatants with IC50 values above 1 μg/mL. A red bar and error bars indicate median and interquartile range. A blue dashed line indicates the highest K_D_ value (2.58 × 10^−8^M) in group 1. The blue number indicates that 94% of group 3 supernatants are above the blue line. (D) Pearson correlation analysis of binding affinities and neutralizing activities of supernatants. Pearson r and p values are shown. (A, C, and D) The gray line at “<10^−12^ M” indicates the measurement limit of the instrument. K_D_ values below the gray line at “10^−10^ M” were considered qualitative instead of quantitative data. (E) Frequency-K_D_ curves of week 39 B cells in the FP-specific (left) or total (right) IgG+ memory B cell population. Apparent K_D_ values were generated on the BG505.DS.SOSIP.S613A trimer. See also Figures S6 and Table S3.

In the example study, a total of 137 supernatants from week 39 PBMCs showed positive binding in FP ELISA (OD_450_ > 0.1) and contained greater than 0.4 μg/mL IgG. These supernatants were selected for trimer binding analysis, as 0.4 μg/mL IgG was sufficient for detecting even low-affinity trimer binding in ELISA (Table S2). 51 supernatants showed no binding (OD450 < 0.1) in an initial ELISA screening of the HIV-1 BG505.DS.SOSIP.S613A trimer, 5 showed no binding in the subsequent Octet analysis, 66 showed apparent K_D_ values between 0.1 and 100 nM using the IgG-Trimer protocol, and 15 showed apparent K_D_ values below 0.1 nM (Figure 5A). Of note, apparent K_D_ values below 0.1 nM were considered qualitative instead of quantitative data; i.e., they bound to the trimer, but the apparent K_D_ values were likely less accurate. This was because the IgG loading concentration (1 nM) was low in the IgG-Trimer protocol, and the overall binding signal was generally low, so an apparent K_D_ below 0.1 nM could be a result of barely detectable dissociation of the analyte.

To further validate the apparent K_D_ values generated using supernatant IgGs, mAbs were recovered from 13 wells with representative supernatant-trimer K_D_ values within the range of 0.1–100 nM. Corresponding Fabs were generated and assessed for binding affinity using the Trimer-Fab protocol. Fab K_D_ values significantly correlated with supernatant IgG K_D_ values (Person r = 0.6080, p = 0.0275) (Figure 5B), and the K_D_ differences were mostly within 5-fold (Figure S6; Table S3). Overall, these results suggested that the apparent binding affinity data from most supernatant IgGs were reliable when they were within the range of 0.1–100 nM.

The 137 supernatants were further categorized into four groups based on their neutralizing activities against the BG505.N611Q virus; groups 1 and 2 included supernatants with IC50 values determined. Specifically, group 1 (N = 53) includes strong neutralizers with an IC50 value below 1 μg/mL, and group 2 (N = 10) includes weak neutralizers with IC50 values above 1 μg/mL. Groups 3 and 4 include supernatants where an IC50 was not reached at the highest tested concentration. Specifically, group 3 (N = 48) includes supernatants with the highest tested IgG concentration above 1 μg/mL, and group 4 (N = 26) includes those with the highest tested IgG concentration below 1 μg/mL. First, the binding affinities with BG505.DS.SOSIP.S613A trimer were compared between groups 1 and 3 supernatants. The highest apparent K_D_ in group 1 supernatants is 2.58 × 10^−8^ M. In contrast, 45 of 48 (94%) group 3 supernatants showed no binding or a K_D_ greater than 2.58 × 10^−8^ M (Figure 5C), suggesting that low affinity to soluble trimer was the main mechanism of non-neutralization in this immunization study. Next, groups 1 and 2 were combined to analyze the binding affinities among neutralizing BCRs. Their apparent K_D_ to the BG505.DS.SOSIP.S613A trimer significantly correlated with the corresponding neutralizing IC50 against the BG505.N611Q virus (Pearson r = 0.4329, p = 0.0019 when supernatant K_D_ values below 0.1 nM were not included; Pearson r = 0.4311, p = 0.0005 when supernatant K_D_ values below 0.1 nM were included) (Figure 5D), suggesting that binding affinity to soluble trimer is also the main contributor to the neutralizing activity against the corresponding virus. Together, these results suggested that the lack of viral membrane in the immunogen design may not be a primary concern for presenting the FP epitope and that further improving BCR affinity to soluble trimer should be one of the prioritized objectives in future vaccine optimization against HIV-1 FP.

### Frequency-K_D_ curves differentiate the frequency and affinity maturation of FP-specific IgG+ memory B cells in different animals

Compared with viral neutralizing activity, which is a common correlate of protection and a main objective of vaccination, binding affinity to the immunogen directly measures the effectiveness of an immunization strategy in driving BCR maturation. Thus, a two-dimensional frequency-affinity curve would directly show the frequency and affinity maturation of antigen-specific B cells. To estimate the frequency of FP-specific IgG+ memory B cells versus their corresponding binding affinities to HIV-1 Env trimer, a similar algorithm (Figure 3A) was applied to generate frequency-K_D_ curves. In this analysis, a K_D_ value of 10^−12^ M was assigned to BCRs showing no detectable dissociation in Octet analysis, and a K_D_ value of 1 M was assigned to non-binding BCRs. The resulting frequency-K_D_ curves differentiated the quantity and trimer affinities of FP-specific IgG+ memory B cells from different animals (Figure 5E). Within the FP-specific IgG+ memory B cell population, the trimer K_D_ values of most cells from animal A11V065 animal were higher than those from other animals, suggesting that they are less affinity matured (Figures 5A and 5E). In total IgG+ memory B cells, overall, the frequency and affinities of FP-specific cells from animal DFPH were higher than those from other animals at a K_D_ cutoff of 1 nM and a frequency cutoff of the top 200 cells in one million IgG+ memory B cells, respectively (Figure 5E, right). Notably, two limitations exist in this example frequency-K_D_ analysis. First, because of the less accurate K_D_ values (<0.1 nM) from a small subset of supernatants, the cell frequency at low K_D_ cutoffs was likely overestimated for animal A11V065 (Figure 5E, right, blue curve). Second, more data points may be needed to refine the analysis for animals 13N008 and A12V098. Together, these results demonstrated that SCAN and frequency-K_D_ analysis can provide highly valuable information by differentiating the frequency and affinity maturation of antigen-specific B cells among different animals at single-cell resolution and suggested further optimization of high-throughput affinity measurement.

### Frequency-IC50 of the dominant neutralizing B cell lineages in immunized animals

**Figure 6.**
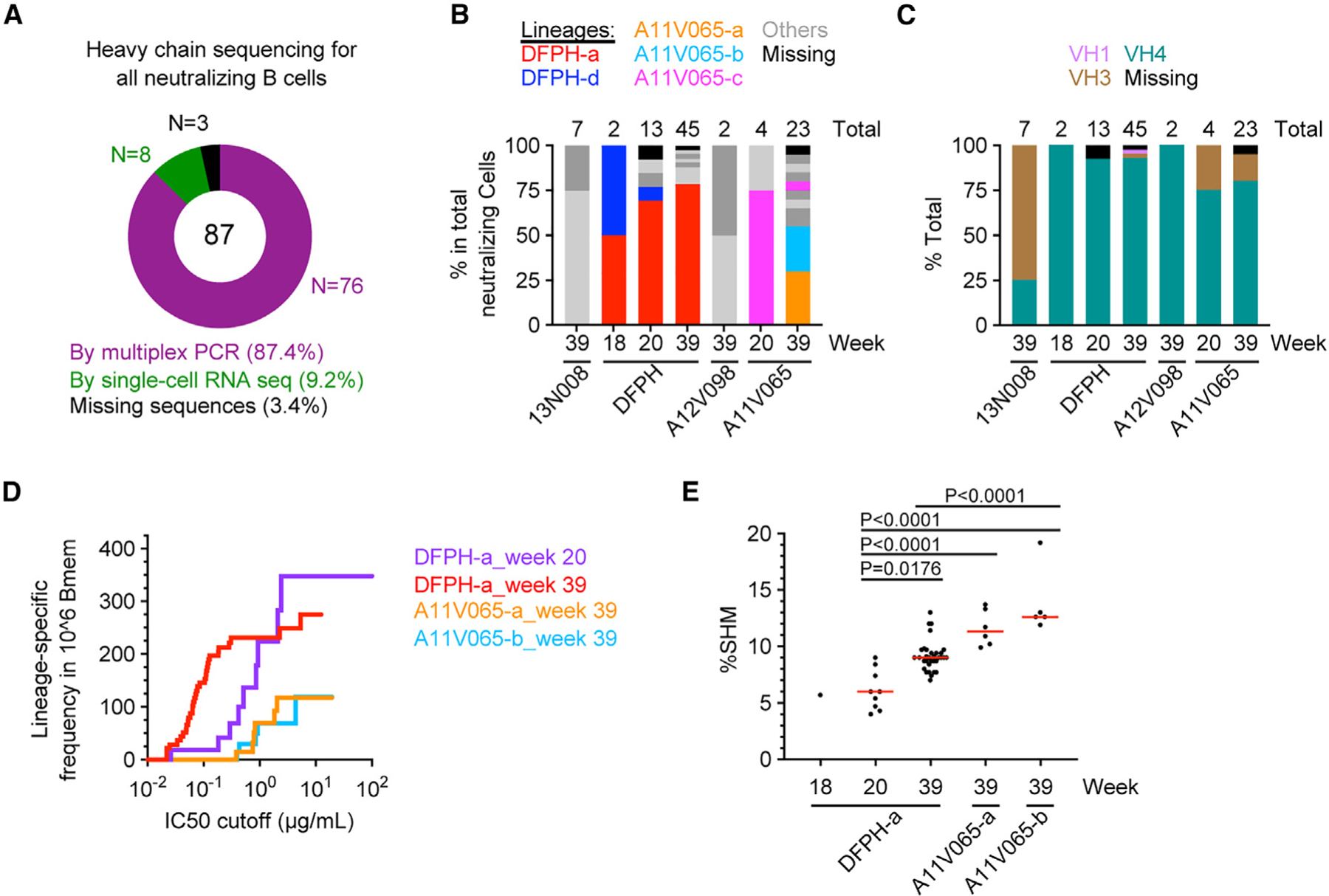
Frequency-potency and affinity maturation of dominant FP-specific neutralizing B cell lineages in immunized NHPs (A) Two-step Ig heavy-chain sequence recovery for all single B cell cultures that neutralized the BG505.N611Q virus. Step 1: multiplex Ig PCR and Sanger sequencing for all wells. Step 2: SMART-based single-cell RNA sequencing for the remaining wells after step 1 recovery. (B) Percentage of each B cell lineage in the total number of neutralizing B cells sampled from the corresponding PBMC sample. The total numbers are shown above each bar graph. Lineages with a size of five or larger (DFPH-a, A11V065-a, and A11V065-b) are colored. Lineages with members identified from multiple time points (DFPH-a, DFPH-d, and A11V065-c) are also colored. Other lineages are shown in dark or light gray, and cells with no sequence recovery are shown in black. (C) Percentage of heavy chain variable domain (VH) family usage in neutralizing B cells. The total numbers of neutralizing B cells from each PBMC sample are shown above each bar graph. VH1, VH3, and VH4 family usage is colored. No VH2 sequence was recovered. Cells with no sequence recovery are shown in black. (D) Frequency-IC50 curves of individual neutralizing lineages in IgG+ memory B cells. Neutralization IC50 values were generated on the BG505.N611Q virus. The B cell lineages and the time points are indicated. (E) Percent somatic hypermutation (%SHM) of neutralizing BCR heavy-chain sequences. A red bar indicates median value. Kruskal-Wallis test (p < 0.0001) and Dunn’s multiple-comparisons test were performed. p values from significant pairs are shown. See also Table S1.

The supernatant functional data from SCAN made it possible to focus the downstream Ig sequence recovery on the B cell population of interest. In the example study, a total of 87 neutralizing supernatants were identified. Using a traditional multiplex PCR approach, Ig heavy-chain sequences were recovered from 76 wells (87.4%) (Figure 6A). 8 additional wells (9.2%) were recovered by SMART-based single-cell RNA sequencing.^29–31^ The high recovery rate (96.6%) was achieved after multicycle trouble shooting, which became possible when effort was focused on a limited number of BCRs and was motivated by their pre-defined potency.

The Ig sequences of top neutralizing BCRs were further analyzed to determine B cell lineages. Because of the close-to-full Ig sequence recovery rate, the resulting B cell lineage distribution is likely a close representation of top neutralizing B cells in the whole IgG+ memory population. Based on 84 heavy-chain sequences, a total of 23 lineages were identified (Table S1), including the previously reported DFPH-a and DFPH-b lineages.^10^ The DFPH-a lineage contributed to 69% and 79% of neutralizing B cells sampled from week 20 and 39 PBMCs from animal DFPH, respectively (Figure 6B). These results definitively concluded that DFPH-a is the only dominant neutralizing lineage in this animal. Such a conclusion could not be made in the previous study because Ig sequences were recovered from only a subset of antigen-specific B cells, and the possibility of primer bias cannot be excluded. Furthermore, VH4 family lineages were the main contributors to FP-directed neutralizing responses in this NHP study (Figure 6C).

To compare the quantity and quality of different B cell lineages or the same lineage from different time points, an adjusted algorithm was established to graph the frequency-IC50 curve for individual lineages. The frequency-IC50 curve of DFPH-a lineage at week 39 is substantially higher than those of the top two lineages from animal A11V065 (A11V065-a and A11V065-b) (Figures 6B and 6D), although the somatic hypermutation rates of A11V065-a and A11V065-b lineage members are generally comparable with or even higher than those of the DFPH-a lineage members (Figure 6E). Furthermore, the frequency-IC50 curves also showed the improved neutralizing potency of the DFPH-a lineage from weeks 20 to 39 (Figure 6D), along with the increased somatic hypermutation level during the same time period (Figure 6E). Together, these findings suggest that DFPH-a is likely a uniquely expanded and matured neutralizing lineage in this study and that future vaccine regimens should aim to elicit similar or more potent lineages more frequently.

### Cross-reactive neutralizing mAbs were efficiently isolated through SCAN

**Figure 7.**
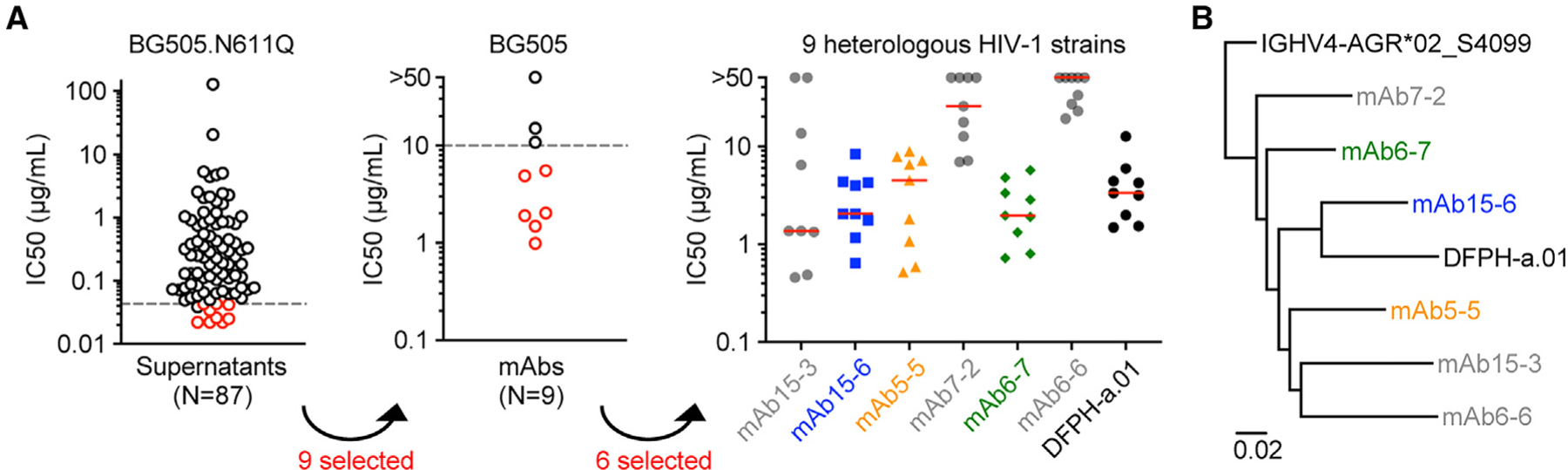
Cross-reactive neutralizing mAbs were efficiently isolated through SCAN (A) Step-by-step identification of cross-neutralizing mAbs. Left: neutralization IC50 values of 87 supernatants against virus BG505.N611Q. A gray line indicates 10 supernatants with the lowest neutralizing IC50 values. Red circles indicate 9 supernatants with mAbs recovered. Center: neutralization IC50 values against autologous wild-type BG505 by 9 selected mAbs. Red circles indicate 6 mAbs with neutralizing IC50 values below 10 μg/mL (gray line). Right: neutralization IC50 values against a panel of 9 multiclade heterologous wild-type HIV-1 strains by 6 selected mAbs and the reference mAb DFPH-a.01. A red bar indicates median value. (B) Maximum-likelihood tree of heavy-chain nucleotide sequences and the germline gene. See also Table S4.

Since the supernatant Ig function data correlate well with the corresponding mAb data (Figures 1C and 1D), SCAN is naturally a promising platform for isolating mAbs with specific function. In the example study, 87 supernatants showed detectable neutralizing IC50 values against virus BG505.N611Q, in which the top 10 potent neutralizers showed an IC50 range of 0.022–0.043 μg/mL (Figures 1B; Table S1). mAbs were recovered from 9 of these 10 top neutralizers. The mAb neutralization titers against BG505.N611Q were highly similar to the corresponding supernatants (Table S4). When tested on an autologous wild-type BG505 virus, 6 mAbs showed IC50 values below 10 μg/mL. When tested on a multiclade panel of 9 heterologous wild-type strains, 3 mAbs (mAb15-6, mAb5-5, and mAb6-7) neutralized all viruses with IC50 values comparable with or even lower than that of the previous best neutralizing mAb DFPH-a.01 (Figure 7A; Table S4). Sequence analysis revealed that all of these 6 mAbs were DFPH-a lineage members from week 39 (Figure 7B; Table S1). Of note, the mAb DFPH-a.01 neutralized 59% of 208 strains, as reported previously.^10^ Together, these results demonstrated that the SCAN workflow isolated cross-neutralizing mAbs with high efficiency.

## DISCUSSION

Using the SCAN workflow, quantitative neutralizing IC50 values were directly generated from single-antigen-specific memory B cell culture supernatant. The resulting data are likely a close representation of the whole circulating IgG+ memory B cells, although unknown bias could still exist and should be monitored in future studies. For example, it is unclear whether different B cell lineages show systematic differences in *in vitro* cell growth and IgG secretion. On top of the quantitative functional data, the two-dimensional frequency-IC50 curves clearly differentiated the neutralizing B cell responses in different samples at single-cell BCR resolution. Additionally, the SCAN workflow is labor and cost efficient. Together, SCAN and frequency-potency analysis are powerful tools for analyzing antigen-specific B cell responses at single-cell resolution, especially when samples from multiple subjects and time points need to be compared.

Additional benefits of SCAN were illustrated in the example study. First, multidimensional functional data could be generated simultaneously, including neutralizing activities against different viral strains, binding activities against different antigen forms, and epitope specificities. Such data could address critical questions. For example, by comparing the supernatant binding affinities to soluble HIV-1 Env trimer and the corresponding neutralizing activities, the BCR affinity to soluble HIV-1 Env SOSIP trimer was demonstrated as the main determinant of vaccine-induced FP-directed neutralizing activities. Second, with quantitative functional data available, the downstream Ig sequencing effort can focus on a limited number of target cells. In the example study, heavy-chain sequences were recovered from 96.6% of the neutralizing B cells. With this high recovery rate, DFPH-a was definitively concluded to be the only dominant neutralizing lineage in animal DFPH. This finding suggested that future immunization designs may need to expand more lineages with similar or higher potency in individual animals. Finally, SCAN can efficiently identify mAbs with functions of interest. DFPH-a.01-like antibodies were identified after sequencing the top 10 B cells, while DFPH-a.01 has been identified previously after sequencing hundreds of sorted cells.^10^

It has been shown that antigen-specific B cells from humans^16,20–23^ and mice^32,33^ can be cultured and that their supernatant IgGs can be used for qualitative functional screening. Using these established or future optimized B cell culture methods, their supernatant IgGs can also be used to generate quantitative functional data and further analyzed by the frequency-potency algorithm. Additionally, antibody functional assays that have been employed in other infectious^3,8,34–37^ and autoimmune^38–42^ diseases could potentially be adapted and plugged into this workflow. Therefore, SCAN and the frequency-potency analysis are expected to be applicable to B cell and antibody analysis in other host species and target pathogens or antigens.

To summarize, SCAN and the frequency-potency analysis have addressed several questions from the previous HIV-1 FP immunization study that were challenging. These findings provide insights for future HIV-1 FP-directed vaccine optimization and recommend these approaches as a general B cell analysis and mAb discovery platform for future targets.

### Limitations of the study

Notably, the single-cell culture recipe can be further improved to increase the frequency of wells with a supernatant IgG concentration above the threshold needed for functional assays. In the example study, functional assays were usually performed on the 10%–20% of wells with supernatant IgG greater than 0.15 μg/mL. More than one vial of PBMCs were used for some samples. Although the sampled cells are expected to be a close representation of the overall IgG+ memory B cell population, a higher recovery rate would still be preferred, especially when the frequencies of antigen-specific cells are low or when PBMC samples are limited. Potential strategies could include using fresh samples, optimizing the sample processing method, optimizing the culture medium recipe, and extending culture time.

## STAR★METHODS

### RESOURCE AVAILABILITY

#### Lead contact

Further information and requests for resources and reagents should be directed to and will be fulfilled by the lead contact, Rui Kong (rui.kong@emory.edu).

#### Materials availability

This study did not generate new unique reagents.

#### Data and code availability

- Data are available within the article or its supplementary materials. Sequences of antibodies have been deposited in NCBI Gen-Bank: OR576798-OR576809.
- This paper does not report original code.
- Any additional information required to reanalyze the data reported in this work paper is available from the lead contact upon request.

### EXPERIMENTAL MODEL AND STUDY PARTICIPANT DETAILS

#### Non-human primate samples

Plasma and PBMC samples from a previously published non-human primate study^10^ were analyzed. The original animal experiments were reviewed and approved by the Animal Care and Use Committee of the Vaccine Research Center, NIAID, NIH. The original animal work was covered under protocol VRC#16–554.

#### Cell lines

HEK293T/17 cells were from ATCC (Cat# CRL-11268). TZM-bl cells were from NIH AIDS Reagent Program (www.aidsreagent.org, Cat# 8129). Expi293F cells were from ThermoFisher Scientific Inc (Invitrogen, Cat# A14528; RRID: CVCL_D615). 3T3muCD40L cells were obtained from Vaccine Research Center, NIAID. The cell lines were cultured following manufacturer suggestions as described in Method Details below.

### METHOD DETAILS

#### Sorting

Non-human primate (NHP) peripheral blood mononuclear cells (PBMCs) were stained with LIVE/DEAD Fixable Violet Stain (ThermoFisher, Waltham, MA) for 10 min at room temperature. Cells were washed with Roswell Park Memorial Institute (RPMI) 1640 Medium (Gibco) with 4% fetal bovine serum (FBS) and incubated with CD3-BV421 (clone SP34–2, BD Biosciences), CD4-BV421 (clone OKT4, BioLegend), CD8-BV421 (clone RPA-T8, BioLegend), CD14-BV421 (clone M5E2, BioLegend), CD20-Cy5.5PerCP (clone 2H7, BioLegend), IgG-A700 (clone G18–145, BD Biosciences), IgM-FITC (clone G20–127, BD Biosciences), FP9-PE, and FP9-APC-Cy7 for 20 min at room temperature. The two FP probes, FP9-PE and FP9-APC-Cy7, were made by conjugating biotinylated nine amino acid HIV-1 fusion peptide (AVGIGAVF) to fluorescence labeled streptavidin and used for staining FP-specific cells. Cells were then washed two times with RPMI with 4% FBS and assessed by flow cytometry. FP double-positive IgG+ memory B cells (CD3^−^CD4^−^CD8^−^CD14^−^CD20+IgG+IgM-FP9+) were sorted into 384-well culture plates at single-cell per well using a FACSAria II sorter (BD Biosciences), and relevant index information recorded. We carefully recorded all cell events during sorting and used the exact numbers of events from the sorter to calculate percentage of probe-stained cells in total IgG+ B cells.

#### Single IgG+ memory B cell culture

Single IgG+ memory B cells were cultured at 37°C in 384-well culture plates (VWR International, Cat# 10814–226). The culture medium was optimized based on a previously reported recipe.^43^ Briefly, Iscove’s Modified Dulbecco’s Medium (IMDM) with GlutaMAX (Gibco) was supplemented with 10% fetal bovine serum (FBS), 0.2% MycoZap Plus-PR (Lonza), 0.8 μg/mL human IL-2 (Kong lab), 0.25 μg/mL human IL-21 (Thermo Fisher), 0.05 μg/mL human IL-4 (Miltenyi Biotec), 0.4 μg/mL human BAFF (GenScript), and 2 μg/mL CpG ODN 2006 (Miltenyi Biotec). Irradiated 3T3muCD40L feeder cells at a concentration of 7500 cells/well were seeded with the single IgG+ memory B cell on day 0. 60 μL of cell culture supernatant was harvested on day 13 or day 21, and then tested for IgG expression and function. The remaining culture containing cells were stored at −80°C for potential Ig sequence recovery.

#### IgG ELISA

All 384-well ELISA plates were coated with mouse anti-human IgG (BD Biosciences, Franklin Lakes, NJ, Cat# 555784) in PBS at 1 μg/mL overnight at 4°C. Coated plates were blocked with B3T buffer (150 mM NaCl, 50 mM Tris-HCl, 1 mM EDTA, 2% bovine serum albumin, 3.3% fetal bovine serum, 0.07% Tween 20, 0.02% Thimerosal) for 1 h at 37°C. After blocking, plates were incubated with 10-fold serially diluted cell culture supernatant starting at 1:20 dilution, or 2-fold serially diluted reference rhesus IgG DFPH-a.01, for 1 h at 37°C. Finally, plates were incubated with mouse anti-monkey IgG conjugated with horseradish peroxidase (HRP) (SouthernBiotech, Birmingham, AL, Cat# 4700–05). All volumes were 25 μL/well except 50 μL/well were used for the blocking step. Plates were washed 6 times between each step with 0.05% Tween 20 in PBS. Plates were developed using 3,3′,5,5′-Tetramethylbenzidine (TMB) (Sera Care, Milford, MA) and read at 450 nM. Supernatant IgG concentrations were interpolated in GraphPad Prism (Version 9.2.0) using reference IgG curves.

#### Fusion peptide ELISA

All 384-well plates were coated with streptavidin in PBS at 2 μg/mL overnight at 4°C. Coated plates were blocked with B3T buffer (150 mM NaCl, 50 mM Tris-HCl, 1 mM EDTA, 2% bovine serum albumin, 3.3% fetal bovine serum, 0.07% Tween 20, 0.02% Thimerosal) for 1 h at 37°C. After blocking, plates were first incubated with FP9-PEG12-biotin^10^ (Genscript, Piscataway, NJ) at 0.4 μM for 1 h at 37°C, then followed with cell culture supernatant at a 1:5 dilution for another hour at 37°C. Finally, plates were incubated with mouse anti-monkey IgG conjugated with horseradish peroxidase (HRP) (SouthernBiotech, Birmingham, AL, Cat# 4700–05). All volumes were 25 μL/well except 50 μL/well was used for the blocking step. Plates were washed 6 times between each step with 0.05% Tween 20 in PBS. Plates were developed using 3,3′,5,5′-Tetramethylbenzidine (TMB) (Sera Care, Milford, MA) and read at 450 nM.

#### HIV-1 Env trimer ELISA

All 384-well plates were coated with Lectin (Sigma, Cat# L8275–5MG) in PBS at 10 μg/mL overnight at 4°C. Coated plates were blocked with B3T buffer (150 mM NaCl, 50 mM Tris-HCl, 1 mM EDTA, 2% bovine serum albumin, 3.3% fetal bovine serum, 0.07% Tween 20, 0.02% Thimerosal) for 1 h at 37°C. After blocking, plates were incubated with BG505.DS.SOSIP.S613A trimer at 2 μg/mL for 1 h at 37°C. Then, cell culture supernatants were assayed at 7-point 5-fold dilutions starting at 1:5 to 1:40 dilutions for another hour at 37°C. Finally, plates were incubated with mouse anti-monkey IgG conjugated with horseradish peroxidase (HRP) (SouthernBiotech, Birmingham, AL, Cat# 4700–05). All volumes were 25 μL/well except 50 μL/well was used for the blocking step. Plates were washed 6 times between each step with 0.05% Tween 20 in PBS. Plates were developed using 3,3′,5,5′-Tetramethylbenzidine (TMB) (Sera Care, Milford, MA, Cat# 5120–0077) and read at 450 nM.

#### Neutralization

HIV-1 Env-pseudotyped virus stocks were generated by cotransfecting 293T cells with a pSG3 Δ Env backbone and an Env expression plasmid as described.^7^ The classic 96-well TZM-bl neutralization assay was performed as described.^7^

To assess single cell culture supernatants for neutralization capabilities in a single round virus infection assay using TZM-bl target cells in 384-well plates, the supernatants were assayed at 7-point 5-fold dilutions starting at 0.6-fold of the undiluted concentration while control monoclonal antibodies (mAbs) were assayed starting at 50 μg/mL. For each well, 7.5 μL of mAbs or supernatants were mixed with 5 μL of diluted HIV-1 Env pseudoviruses and incubated for 30 min at 37°C. TZM-bl cells were resuspended at a concentration of 500,000 cells/mL with 0.07 mg/mL of DEAE-Dextran hydrochloride (Millipore Sigma, Burlington, MA) in PBS. For each well, 5 μL of TZM-bl cell suspension was added to the antibody-virus mixture and incubated for 2 days at 37°C. On day 3, cells were lysed and incubated with Steadylite plus (PerkinElmer, Waltham, MA) for 20 min at 37°C. Plates were then read on a luminometer and luciferase activity was measured. A nonlinear regression dose-response curve was fitted using an asymmetric sigmoidal curve with 5-parameters. The 50%, 80%, and 90% inhibitory concentrations (IC50, IC80, and IC90) were determined for each supernatant. Of note, if 50% inhibition was not achieved, it suggested that the supernatant was either not neutralizing or the neutralizing IC50 concentration was higher than the tested starting concentration.

A panel of 9 heterologous viruses was selected to evaluate the cross-reactivity of HIV-1 fusion peptide-directed neutralization. The 9 viruses in this panel were selected from a multi-clade panel of 208 HIV-1 strains^7^ using the following criteria: 1) the sequence of eight N-terminal amino acids (AVGIGAVF) of the fusion peptide matched the FP sequence of immunogens used in the NHP study; 2) the viral sequences showed a complete glycosylation profile surrounding the fusion peptide epitope. Specifically, N-linked glycosylation sites are observed at the amino acid positions of 88, 241, 448, 611, 625, and 637; 3) the panel includes representative strains from multiple clades A, B, C, AC, AD, and BC (Table S4); 4) all strains are resistant to V3 and CD4i antibodies (17b, 48d, F105, 3074, 447–52D). Together, this is a multi-clade panel of strains with fusion peptide sequence matching the immunogen, and it has excluded strains that are generally sensitive to V3 and CD4i antibodies or potentially super-sensitive to fusion peptide antibodies due to the lack of glycosylation.

#### Frequency-potency curve

We used the Kaplan-Meier estimation of survival in GraphPad Prism (Version 9.2.0) to analyze the frequency-potency of IgG+ memory B cells in the PBMC samples. As we described above, probe-specific IgG+ memory B cells were sorted at a single cell per well and cultured for 2 or 3 weeks. The wells with supernatant IgG concentration greater than 0.15 μg/mL and FP ELISA OD450 greater than 0.1 represent the antigen-specific IgG+ memory B cell population (Figure 2A). These supernatants were then serially diluted and tested for HIV-1 neutralization. If 50% inhibition of virus entry was achieved, the IC50 value was determined. If it was not achieved, the highest tested concentration was recorded. Of note, the highest tested concentration was different for each supernatant as described above. According to the neutralization protocol, the starting (highest tested) concentration of each supernatant is the corresponding IgG concentration multiplied by 0.6. For the Kaplan-Meier analysis, the event of interest was defined as achieving 50% inhibition. Supernatants that did not reach 50% inhibition at the highest tested concentration were censored. Thus, an IC50 value was recorded as an observation for the event of interest, and the highest tested concentration was recorded as a censored observation. The resulting Kaplan-Meier survival curve shows an estimation of the frequency-potency of IgG+ memory B cells in the antigen-specific population (Figure 3A).

To further estimate the frequency-potency in the total IgG+ memory B cell population, we first calculated two factors for each PBMC sample. The first factor was the percentage of probe-positive cells in total IgG+ memory B cells in FACS. The second factor was the percentage of wells with FP ELISA OD450 greater than 0.1 in all wells with IgG concentrations greater than 0.15 μg/mL, representing the frequency of true FP-specific cells in the probe-positive population. These two steps were the only instances that enriched antigen-specific cells by screening out probe-negative cells and cells that were false positively stained by probes (Figure 2A). We then used these two factors and the generated frequency-potency table of the antigen-specific population to estimate the frequency in total IgG+ memory B cells. To do so, each frequency value was multiplied by the two factors (Figure 3A). The resulting frequency-potency table was used to graph the frequency-potency curve in total IgG+ memory B cells.

To graph the frequency-IC50 curve for individual B cell lineages, the IC50 values contributed by supernatants from other lineages were now considered as censored observations, and only IC50 values contributed by the target lineage members were kept as events of interest.

#### Bio-layer interferometry

Binding kinetics were measured using the Octet RED384 system (ForteBio). The assay was performed in 384-well tilted bottom microplates (ForteBio, Cat# 18–5080) at 25°C, and the total working volume for samples and buffer was more than 45 μL per well. Prior to each assay, new sensor tips were pre-hydrated in 1% BSA PBS for at least 10 min, followed by equilibration with 1% BSA PBS for 60 s. The sensor tips were first loaded with ligands and then were dipped into the wells containing analytes followed by new wells containing fresh 1% BSA PBS for the association and dissociation steps, respectively. The sensor tips were dipped into 1% BSA PBS wells for 60 s between all steps except the association/dissociation steps, and the plate was agitated at 1,000 rpm over the entire course of the experiment. All samples were prepared to contain 1% BSA in PBS.

To measure the binding kinetics between IgG and Trimer (IgG-Trimer method), anti-mouse Fc capture (AMC) biosensor tips (ForteBio, Cat# 18–5089) were dipped into the wells containing 50 nM anti-human mouse IgG (BD Biosciences, Cat# 555784) for 1,000 s, followed by 1 nM mAb or supernatant containing 1 nM IgG until the signal reached 0.25 nm for this step. 100–800 nM of the Trimer was used as an analyte, and the binding interactions were monitored for 1,000 s of the association step followed by 1,500 s of the dissociation step.

To measure the binding kinetics between Trimer and Fab (Trimer-Fab method), anti-human Fc capture (AHC) biosensor tips (ForteBio, Cat# 18–5063) were dipped into the wells containing 25 nM RM19R-human Fc IgG^44^ for 420 s followed by 100 nM Trimer for 420 s. Five to seven serially diluted concentrations per Fab, starting at 50–800 nM, were used as analytes. The binding interactions were monitored for 60–3000 s of the association step followed by 40–3600 s of the dissociation step.

Bio-layer interferometry data were analyzed using ForteBio Octet Data Analysis HT software version 12.0.2.59. Sensorgrams were corrected with blank reference curves and smoothed by a Savitzky-Golay filter. For IgG-Trimer method, sensorgram of a single concentration was fitted locally to a 1:1 Langmuir model. The window of interest was usually set as 0–50 s and 0–700 s for the association and dissociation steps, respectively. For Trimer-Fab method, sensorgrams of more than four concentrations were fitted globally to a 1:1 Langmuir model. The window of interest was set to encompass the full association and dissociation time period.

#### Ig sequence recovery by multiplex PCR

Frozen cell cultures were lysed with 0.29 μL of 1M Tris-HCl pH8 (Quality Biological, Cat# 351-007-101), 0.25 μL of RNAse inhibitor (NEB, Cat# M0314L), and 19.46 μL of molecular grade water. cDNA was generated in a 25 μL reverse transcription mixture containing 1 μL of Superscript IV reverse transcriptase (200U/μl, Invitrogen, Cat# 18090050), 5 μL of 5X first strand buffer, 3 μL of random hexamer (2μM), 2 μL of dNTP mix (10mM), 1.25 μL of DTT, 0.0625 μL of IgePAL, 0.5 μL of RNase OUT (Invitrogen, Cat# 10777019), and about 12 mL of cell lysate, using the following program: 10 min at 42°C, 10 min at 50°C, 10 min at 55°C, 10 min at 80°C. Ig heavy and light chain sequences were recovered by nested PCR using 5′ primers that were previously published^19^ and 3′ primers that were newly designed. The new primers include two heavy chain 3′ primers 3HuRMCgCH1_78–101 (5′-GGGAAGTAGTCCTTGACCAGG CAG-3′) and 3HuRMCgCH1_73–95 (5′-TAGTCCTTGACCAGGCAGCCCAG-3′), and two kappa chain 3′ primers 3HuRMCK_76–103 (5′-TGGGATAGAAGTTATTCAGCAGGCACAC-3′) and 3HuRMCK_69–95 (5′-AAGTTATTCAGCAGGCACACAACAGAG-3′). Trouble shooting PCRs were performed for a subset of cell lysates using primers designed specifically for individual lysates.

#### Ig sequence recovery by SMART-based single-cell RNA sequencing

5 μL frozen cell lysates from single B cell culture were thawed on ice. RNA was purified using RNACleanXP Solid Phase Reversible Immobilization (SPRI) beads (Beckman Coulter). The beads with bound RNA were re-suspended in Clontech buffers for mRNA amplification using 5′ template switching PCR with the Clontech SMART-Seq v4 Ultra Low Input RNA kit according to the manufacturer’s instructions. Amplified cDNA was fragmented and attached with dual-indexed barcodes using Illumina Nextera XT DNA Library Prep kits. Libraries were validated on an Agilent 2100 Bioanalyzer and 4200 TapeStation and also quantified on a Qubit 4 Fluorometer with 1X dsDNA HS Assay Kit. Libraries were then pooled and sequenced with 200 cycle kits and a read depth of 1 million reads/sample on an Illumina MiSeq v3 or NovaSeq 6000 S4 flow cell. Sequencing was conducted at the Emory National Primate Research Center Genomics Core Laboratory (http://www.yerkes.emory.edu/nhp_genomics_core).

The BALDR pipeline^45^ was used for the reconstruction of immunoglobulin heavy and light chains using the FIlterNonIG method for rhesus macaques. The pipeline used Trimmomatic v0.36^46^ for trimming reads with the parameters: ILLUMINACLIP: adapter_file:2: 30:10 LEADING:3 TRAILING:3 SLIDINGWINDOW:4:20 MINLEN:36. STAR v2.5.2b^47^ was used to align reads to the MacaMv7 genome ref. 48. Only reads that did not map to any annotated gene were filtered using samtools v1.3.1^49^ and seqtk v1.2 and assembled using Trinity v2.3.2.^50^ The assembled sequences were annotated using IgBLAST v 1.6.1.^51^ The number of reads mapping to the reconstructed Ig sequences was determined using bowtie v2.3.0.^52^ The final sequences for each cell were manually selected from the BALDR output.

#### B cell lineage analysis

Germline genes and CDR regions were assigned for each antibody sequence using SONAR.^53^ Antibody lineages were identified by clustering sequences with 70% CDRH3 identity using USEARCH.^54^ The germline gene database was obtained from our previous study.^10^

#### Phylogenetic analysis

Sequence alignment was performed in Geneious Prime 2022.1.1 using MUSCLE 3.8.425. Maximum likelihood analysis was performed in Geneious Prime 2022.1.1 using PhyML 3.3.20180621. Phylogenetic tree was made in FigTree v1.4.4.

#### IgG and fab production

Antibody heavy and light chain variable domain sequences were cloned into expression vectors that contain rhesus macaque immunoglobulin constant regions. IgG was expressed in Expi293F cells by cotransfecting heavy and light chain plasmids and purified using AmMag Protein A Magnetic Beads (GenScript, Cat# L00695) according to the manufacturer’s instructions. To produce Fab, IgG proteins carrying a HRV3 cleavage site in the heavy-chain hinge region were cleaved by HRV3C. Fab fragments were purified by removing Fc-containing proteins using AmMag Protein A Magnetic Beads (GenScript, Cat#L00695).

### QUANTIFICATION AND STATISTICAL ANALYSIS

For all sorted cells, univariable logistic regression was used to test if positive (>0.15 μg/mL) or negative (<0.15 μg/mL) IgG expression was affected by B cell receptor staining. Specifically, the compensated fluorescence intensity values for IgG-Alexa 700, FP9-PE, and FP9-Cy7-APC staining were gathered from index sorting statistics for each sorted cell. Supernatant IgG concentration was determined for each cell as described above. Using the fluorescence intensities of each sorted cell and their corresponding supernatant IgG concentrations, a univariable logistic model was created on GraphPad Prism (Version 9.2.0) using a binary outcome variable. For the analysis, a successful outcome was defined as a positive IgG expression (>0.15 μg/mL). A p value greater than 0.05 indicated no association between the IgG expression status and B cell receptor staining.

For cells with supernatant IgG concentration above 0.15 μg/mL, univariable linear regression was performed in GraphPad Prism (Version 9.2.0) to test if IgG concentration level was associated with B cell receptor staining. Fluorescence intensity values were gathered as described above. A p value greater than 0.05 indicated no association between the IgG concentration level and B cell receptor staining.

The above logistic and linear regression analyses were carried out for all 14 SCAN experiments. After that, the results were combined using random-effects meta-analyses from the metafor R package (https://doi.org/10.18637/jss.v036.i03).

## Supplementary Material

1

## Figures and Tables

**Table:** KEY RESOURCES TABLE

REAGENT or RESOURCE	SOURCE	IDENTIFIER
Antibodies		
LIVE/DEAD^™^ Fixable Violet Stain	ThermoFisher Scientific	Cat# L34955
Anti-human CD3 BV421	BD Biosciences	Cat# 562877; RRID:AB_2737860
Anti-human CD4 BV421	BioLegend	Cat# 317433; RRID:AB_11150413
Anti-human CD8 BV421	BioLegend	Cat# 301036; RRID:AB_10898322
Anti-human CD14 BV421	BioLegend	Cat# 301830; RRID:AB_10959324
Anti-human CD20 PerCP/Cy5.5	BioLegend	Cat# 302326; RRID:AB_893285
Anti-Human IgM FITC	BD Biosciences	Cat# 562029; RRID:AB_10895795
Anti-Human IgG A700	BD Biosciences	Cat# 561296; RRID:AB_10612406
FP9 PE	This paper	N/A
FP9 APC-Cy7	This paper	N/A
DFPH-a.01	Kong et al.^10^	N/A
Mouse anti-human IgG	BD Biosciences	Cat# 555784; RRID:AB_396119
FP-specific mAbs from immunized NHP	Kong et al.^10^ and this paper	N/A
Bacterial and virus strains		
BG505 N611Q mutant	Kong et al.^14^	N/A
HIV-1 wild-type strains	Kong et al.^14^	N/A
Biological samples		
NHP plasma and PBMCs	Kong et al.^10^	N/A
Chemicals, peptides, and recombinant proteins		
FP9-PEG12-biotin	Genscript	N/A
Mouse Anti-Monkey IgG-HRP	SouthernBiotech	Cat# 4700–05
Streptavidin, R-PE conjugate	ThermoFisher Scientific	Cat# S21388
Streptavidin, APC-Cy7 conjugate	BD Biosciences	Cat# 554063
Streptavidin	VWR	Cat# 97062–808
SureBlue TMB Microwell Peroxidase Substrate	VWR	Cat# 95059–286
DEAE-Dextran hydrochloride	Sigma Aldrich	Cat# D9885–10G
Human IL-2	This paper	N/A
Human IL-21	ThermoFisher Scientific	Cat# PHC0213
Human IL-4	Miltenyi Biotec	Cat# 130-093-922
Human BAFF	GenScript	Cat# Z02976–1
HIV-1 BG505.DS.SOSIP.S613A Env trimer	This paper	N/A
Critical commercial assays		
PEI 25K transfection reagent	Polysciences	Cat#23966–1
CTS Opti-MEM I Medium	Fisher Scientific	Cat# A4124802
Steadylite plus Reporter Gene Assay System	PerkinElmer	Cat# 6066751
Deposited data		
Sequence of antibodies	This paper	GenBank: OR576798-OR576809
Experimental models: cell lines		
3T3muCD40L cells	NIH/VRC	N/A
TZM-bl cells	NIH AIDS Reagent Program	Cat# 8129
Expi293 cells	ThermoFisher Scientific Inc	Cat# A14527
Experimental models: organisms/strains		
Indian origin rhesus macaque	Kong et al.^10^	N/A
Oligonucleotides		
CpG ODN 2006	Miltenyi Biotec	Cat# 130-100-105
Recombinant DNA		
Rhesus Macaque Igg expression vector	NIH/VRC, Mason et al.^19^	VRC3950
Rhesus Macaque Igk expression vector	NIH/VRC, Mason et al.^19^	VRC3951
Rhesus Macaque Igl expression vector	NIH/VRC, Mason et al.^19^	VRC3952
pVRC8400 vector	https://www.addgene.org	Cat# 63160
Software and algorithms		
GraphPad Prism 9.0.0 Software	GraphPad Prism Software	N/A
FlowJo v10.8.1	FlowJo	N/A

## Highlights

- Frequency-potency of antigen-specific memory B cells at single-cell resolution
- Affinities to soluble trimer dominate vaccine-induced, FP-directed neutralization
- 96% Ig sequence recovery definitively demonstrates dominant neutralizing lineages
- SCAN efficiently identifies cross-neutralizing monoclonal antibodies
