# Supplementary material for: Frequency-potency analysis of IgG+ memory B cells delineates neutralizing antibody responses at single-cell resolution
Source: Cell Rep. Author manuscript; Available in PMC 2024 Apr 9. (PMC11003769; doi:10.1016/j.celrep.2024.113948)
Supplement: 1 [file NIHMS1980969-supplement-1.pdf]

Cell Reports, Volume *43*

Supplemental information

Frequency-potency analysis of IgG+ memory B cells delineates neutralizing antibody responses at single-cell resolution

Michelle K. Tenggara, Seo-Ho Oh, Catherine Yang, Hardik K. Nariya, Amanda M. Metz, Amit A. Upadhyay, Dedeepya R. Gudipati, Lizheng Guo, Emily G. McGhee, Kiran Gill, Elise G. Viox, Rosemarie D. Mason, Nicole A. Doria-Rose, Kathryn E. Foulds, John R. Mascola, Yuhong Du, Haian Fu, John D. Altman, Qi Yan, Zizhang Sheng, Steven E. Bosinger, and Rui Kong

Figure S1

A

Protocol	Mixture		Sample dilution		Sample needed	
	Sample (μL)	Virus (μL)	Starting dilution	Serial dilution	/reaction (μL)*	/duplicate (μL)*
**Previous**						
96-well	10	40	1:5	1:5	12.5	25
384-well	12	48	1:5	1:5	15	30
**Optimized**						
384-well	7.5	5	1:1.67	1:5	9.375	18.75

* Extra volume needed for loss during liquid transfer was not included.

B

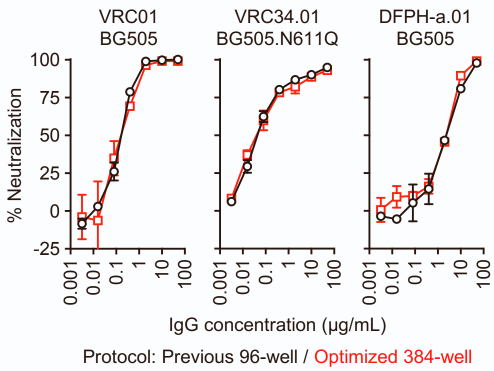

Figure S1. Optimized 384-well HIV-1 neutralization assay. Related to Figure 1. (**A**) Comparison of starting dilution factor and sample usage among HIV-1 neutralization assay protocols. After optimization, both the starting dilution factor and the needed sample volume are lower. Of note, usually a total of 60 ul of supernatant are obtained from the culture. (**B**) Neutralization curves generated by previous 96-well protocol and optimized 384-well protocol. Curves from three pairs of reference mAb and virus are shown, with mAb/virus indicated above the curve. Mean and SD are shown for duplicated wells.

Figure S2

A

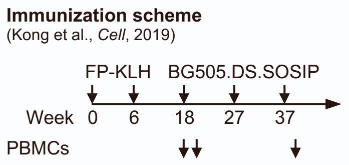

B

Subject	Week	Sorted	IgG conc. >0.15 μg/mL	FP ELISA OD450>0.1	BG505.N611Q neutralization IC50 determined
13N008	18	11	0	0	0
	20	121	14	10	0
	39	121	8	7	4
DFPH	18	51	9	8	2
	20	330	58	52	13
	39	278	67	52	42
A12V098	18	5	0	0	0
	20	119	12	12	0
	39	88	9	9	2
A11V065	18	19	2	2	0
	20	150	33	29	4
	39	528	117	102	20
Total		1821	329	283	87

C

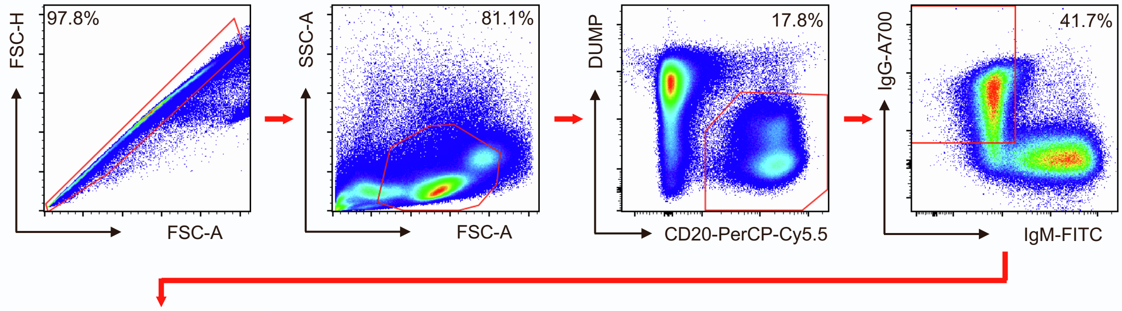
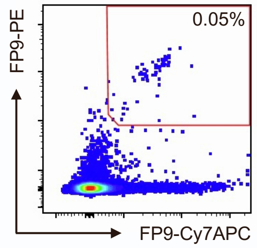

D

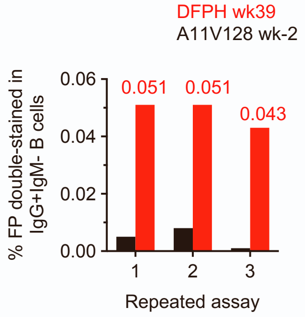

E

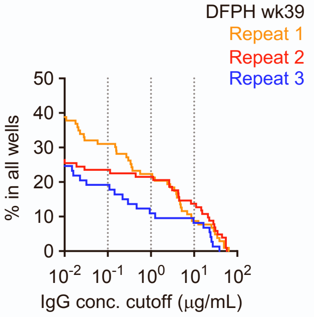

Figure S2. An example analysis by SCAN. Related to Figure 1. (**A**) Immunization scheme of the previous NHP immunization study that was analyzed by SCAN. (**B**) Summary of the analysis. 12 PBMC samples from 4 NHPs and 3 time points were analyzed. FP probe positive cells were sorted. Supernatants with IgG concentration greater than 0.15 μg/mL were tested in FP ELISA. Supernatants showing positive binding in FP ELISA (OD450>0.1) were further tested in neutralization assay. Number of wells are shown for each step. (**C-E**) PBMC sample from animal DFPH at week 39 post immunization were sorted, cultured, and analyzed in three independently repeated assays (repeat 1-3). (**C**) Probe staining of DFPH week39 CD20+IgG+IgM- B cells. Cells with double probe staining were sorted and their frequency is shown. A representative of three repeated assays is shown. Notably, non-specific binding to FP9-Cy7APC probe was observed, suggesting future optimization using other fluorophores. (**D**) Percent frequency of FP double-stained cells in IgG+IgM- B cells from animal DFPH at week 39 post immunization (red) and animal A11V128 at week -2 prior to immunization (black). (**E**) Percentage-concentration curve showing the frequency of total culture wells with supernatant IgG concentration higher than the cutoff value.

Figure S3

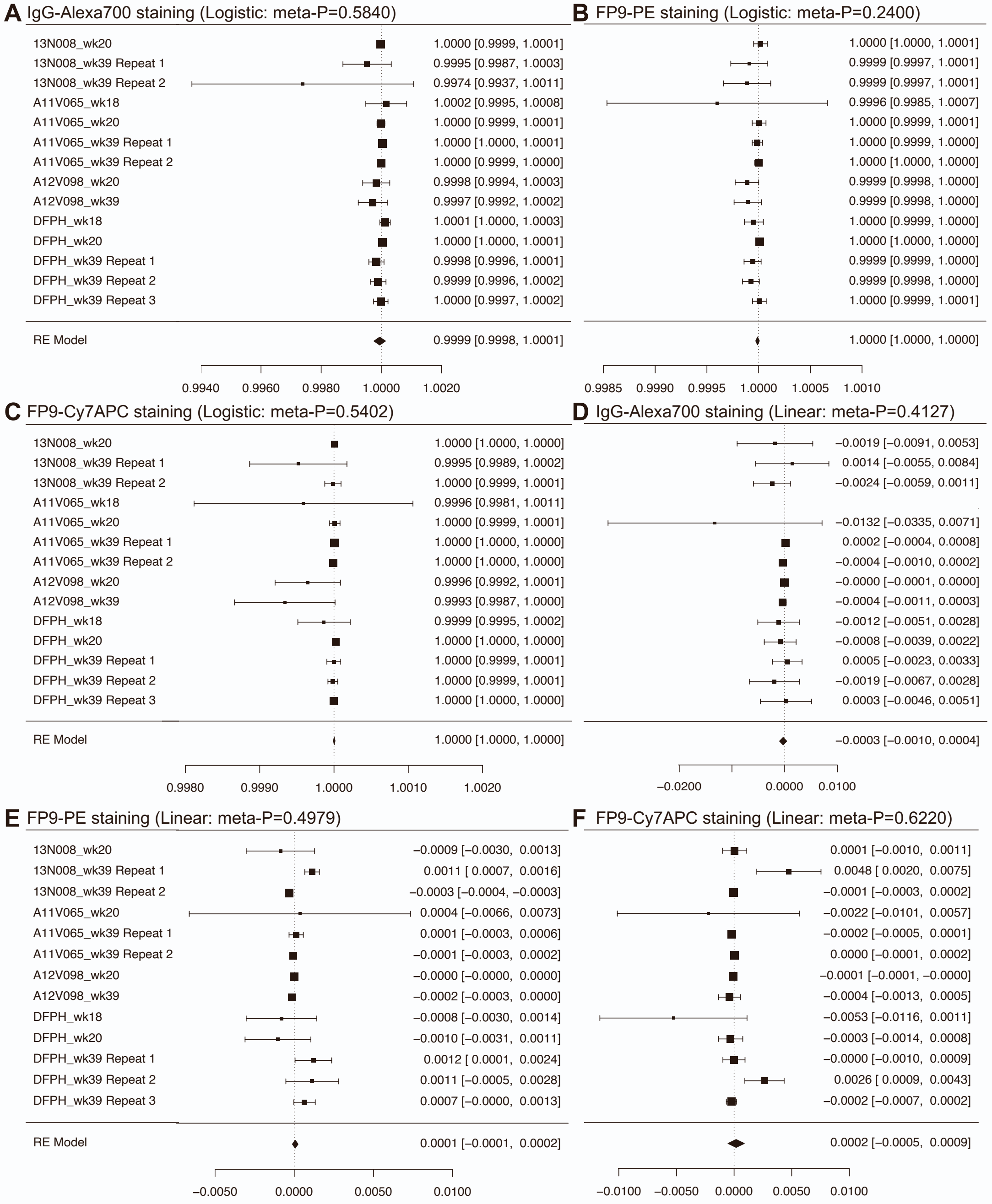

Figure S3. Meta-analyses demonstrated that IgG concentration in single B cell culture supernatant is unrelated to BCR staining in flow cytometry. Related to Figure 2. (**A-C**) Logistic analysis was performed to test if BCR staining in sorting impacts whether supernatant IgG concentration is above or below 0.15 μg/mL. (**D-F**) Linear regression was performed to test if BCR staining impacts the level of IgG concentration when it was above 0.15 μg/mL. (**A-F**) Results from individual data sets and meta-analyses are shown as forest plots. The meta-analyses were conducted using random effect (RE) model. The black squares represent the odds ratio (OR) (panel A-C) or the effect size (panel D-F). The whiskers show the corresponding 95% confidence intervals. P values are shown for each meta-analysis.

Figure S4

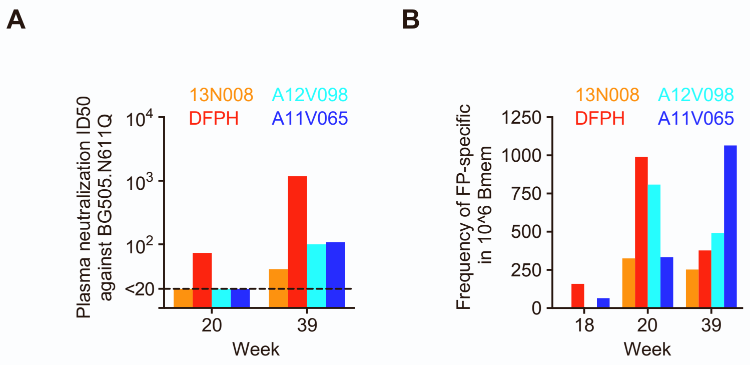

Figure S4. Plasma neutralization titers and frequency of total FP-specific memory B cells in immunized NHPs. Related to Figure 3. (**A**) Week 20 and 39 plasma neutralization ID50 titers against HIV-1 BG505.N611Q virus. (**B**) Frequency of FP-specific cells in memory B cells in week 18, 20, and 39 PBMC samples. Of note, the frequency was estimated by the following formula to exclude false-positive hits in flow cytometry: Frequency of FP-specific cells in IgG+ B = % FP probe stained in IgG+ B cells in flow cytometry X % Positive binding wells in FP ELISA.

Figure S5

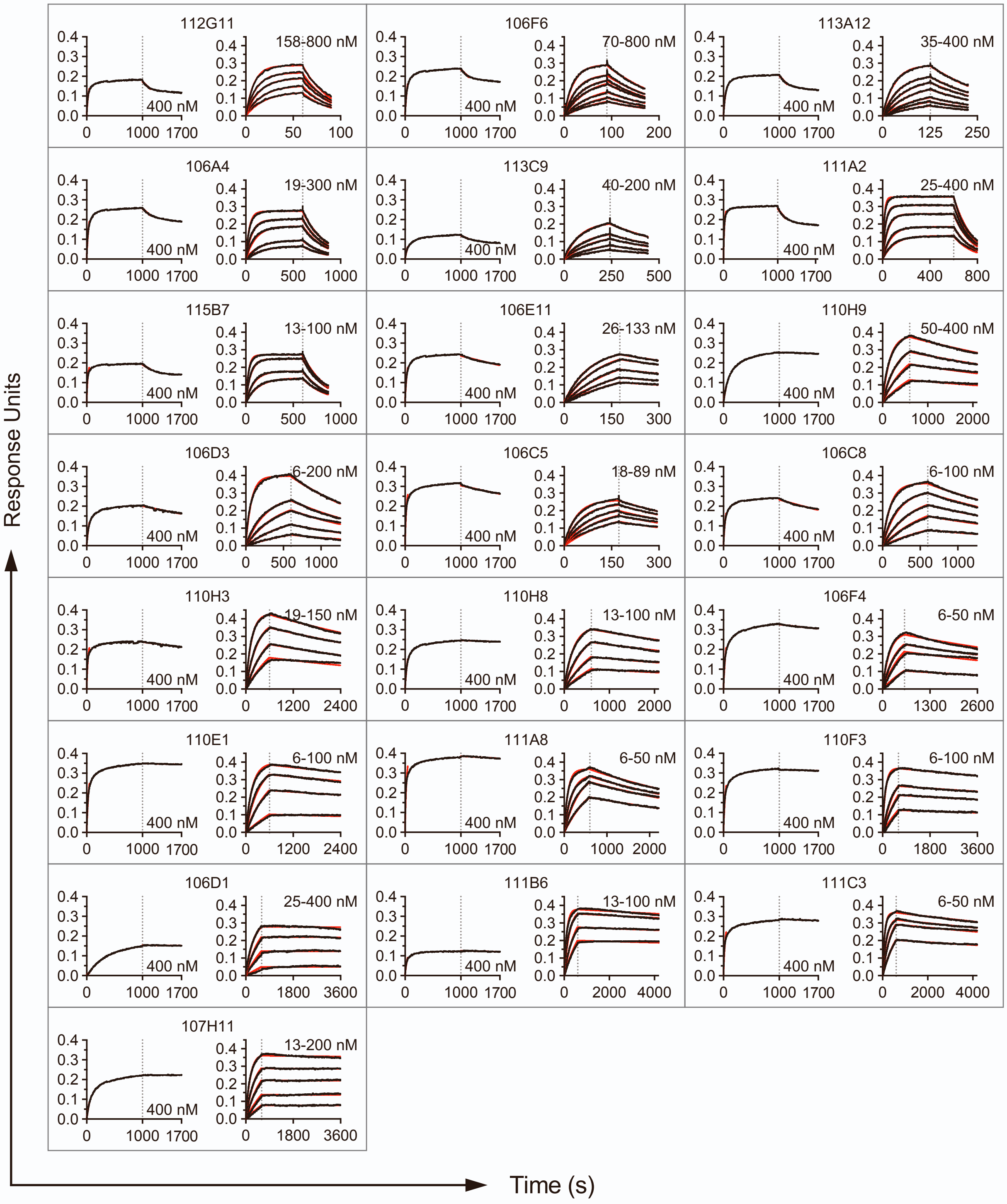

Figure S5. Binding kinetics of reference mAbs and corresponding Fabs to BG505.DS.SOSIP.S613A trimer. Related to Figure 4. For each mAb, binding kinetics were measured in Octet using IgG-Trimer protocol (left) or Trimer-Fab protocol (right). Black lines indicate background subtracted response data and red lines indicate fit of each data set to a 1:1 Langmuir binding model. Concentrations of the analyte are shown. KD, ka, and kd values are reported in Table S2.

Figure S6

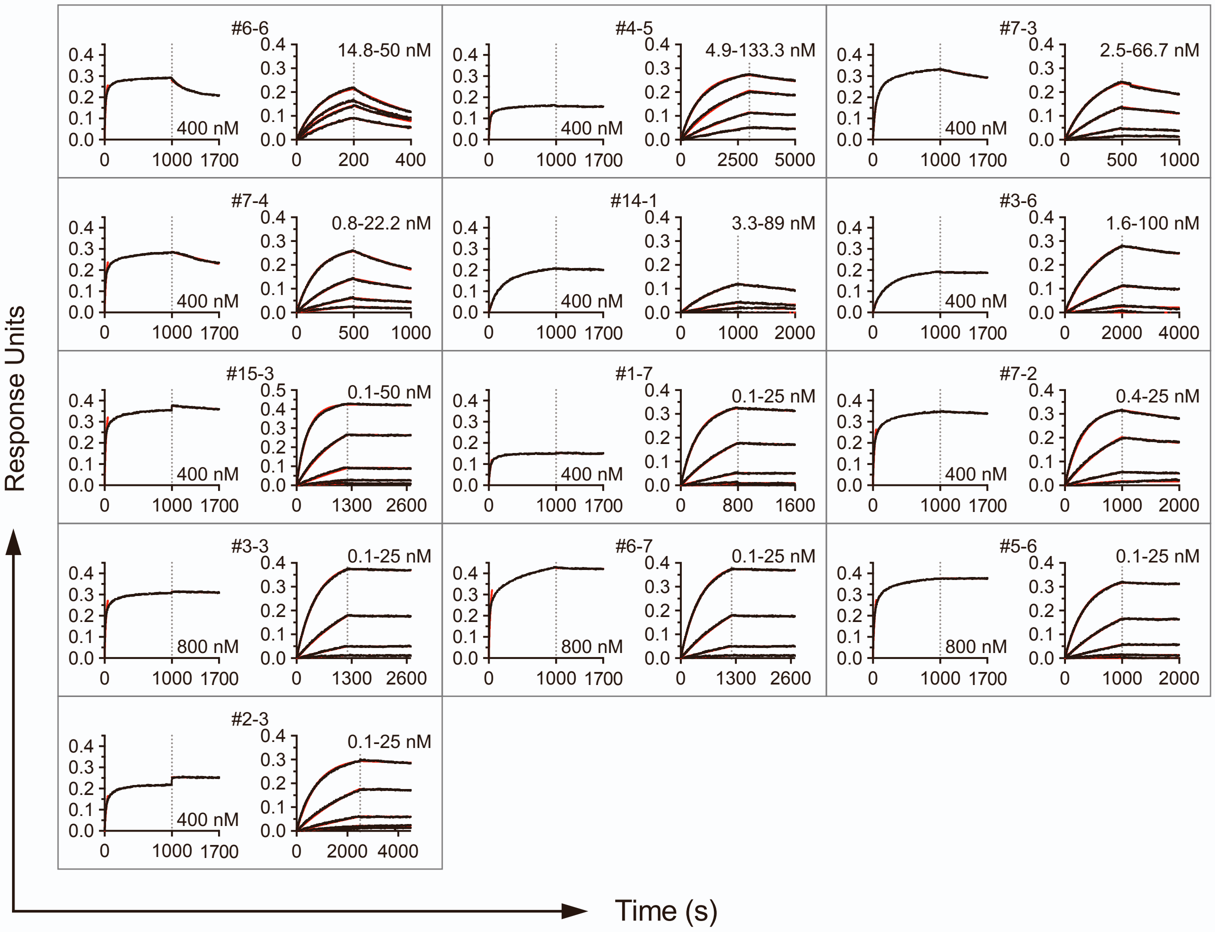

Figure S6. Binding kinetics of supernatant IgGs and corresponding Fabs to BG505.DS.SOSIP.S613A trimer. Related to Figure 5. Binding kinetics of supernatant IgGs were measured in Octet using IgG-Trimer protocol (left). Binding kinetics of corresponding Fab were measured using Trimer-Fab protocol (right). Black lines indicate background subtracted response data and red lines indicate fit of each data set to a 1:1 Langmuir binding model. Concentrations of the analyte are shown. KD, ka, and kd values are reported in Table S3.

Table S1A

Table S1. Neutralization IC50s of single B cell culture supernatants and the corresponding antibody heavy chain sequence analysis. Related to Figures 1, 3, 6, and 7. A total of 87 supernatants from animal DFPH and other animals are shown. Heavy chain sequences were aligned on individualized IgDiscover database. The origin of the B cells, including animal name and time point, are shown. Supernatant neutralizing IC50s against BG505.N611Q virus are shown. Lineage ID was assigned to key lineages.

Table S1A. Supernatants from animal DFPH.

Table S1B. Supernatants from animals 13N008, A12V098, and A11V065.

Culture supernatant				Heavy chain sequencing and analysis							
ID	Animal	Week	BG505.N611Q IC50 (μg/mL)	Method	Clone size	VH	VJ	%SHM	CDR3 length	CDR3 sequence	Lineage ID
12-3	DFPH	18	0.558	Multiplex PCR	43	IGHV4-AGR*02_S4099	IGHJ5-1*01	5.7	20	CSSRAKIYHSGSYSSGGRIDVW	DFPH-a
10-5	DFPH	20	0.295	Multiplex PCR	43	IGHV4-AGR*02_S4099	IGHJ5-1*01	6	20	CTSRAKVHSSASYYYGGRIDVW	DFPH-a
10-6	DFPH	20	0.423	Multiplex PCR	43	IGHV4-AGR*02_S4099	IGHJ5-1*01	4.7	20	CSSRAKIYSGGSYSSGGRIDVW	DFPH-a
10-8	DFPH	20	0.510	Multiplex PCR	43	IGHV4-AGR*02_S4099	IGHJ5-1*01	6	20	CSSRAKIYSGGSYSSGGRIDVW	DFPH-a
11-1	DFPH	20	0.026	Multiplex PCR	43	IGHV4-AGR*02_S4099	IGHJ5-1*01	4	20	CSSRAKIYYSASYYYGGRIDVW	DFPH-a
11-2	DFPH	20	0.183	Multiplex PCR	43	IGHV4-AGR*02_S4099	IGHJ5-1*01	5.4	20	CTSRAKIYHGGGYYSGGRIDVW	DFPH-a
11-5	DFPH	20	0.936	Multiplex PCR	43	IGHV4-AGR*02_S4099	IGHJ5-1*01	7.4	20	CTTRAKVFSSGSYYSGGRIDVW	DFPH-a
11-6	DFPH	20	2.118	Multiplex PCR	43	IGHV4-AGR*02_S4099	IGHJ5-1*01	8.4	20	CTSRAKVYSTGSYYSGGRIDVW	DFPH-a
11-7	DFPH	20	2.415	Multiplex PCR	43	IGHV4-AGR*02_S4099	IGHJ5-1*01	9	20	CTSRAKVYSTGSYYSGGRIDVW	DFPH-a
12-1	DFPH	20	0.858	Multiplex PCR	43	IGHV4-AGR*02_S4099	IGHJ5-1*01	4.3	20	CSSRAKIYYSASYYYGGRIDVW	DFPH-a
1-1	DFPH	39	0.050	Multiplex PCR	43	IGHV4-AGR*02_S4099	IGHJ5-1*01	9	20	CTSRAEVFFSVGYHSGRRIDVW	DFPH-a
1-2	DFPH	39	0.111	Multiplex PCR	43	IGHV4-AGR*02_S4099	IGHJ5-1*01	7.7	20	CTSRAKVHTSASYYYGGRIDVW	DFPH-a
1-4	DFPH	39	0.115	Multiplex PCR	43	IGHV4-AGR*02_S4099	IGHJ5-1*01	8	20	CASRAKIYYSPGHDTGGRIDVW	DFPH-a
1-5	DFPH	39	0.049	Multiplex PCR	43	IGHV4-AGR*02_S4099	IGHJ5-1*01	9	20	CTSRAKIYSSGTYYSGGRIDVW	DFPH-a
1-6	DFPH	39	0.069	Multiplex PCR	43	IGHV4-AGR*02_S4099	IGHJ5-1*01	8.7	21	CSSRAKIYTGGGSYSSEGRIDVW	DFPH-a
2-2	DFPH	39	0.058	Multiplex PCR	43	IGHV4-AGR*02_S4099	IGHJ5-1*01	8.7	20	CTSRAKVHYSASSYYGGRIDVW	DFPH-a
2-3	DFPH	39	0.072	Multiplex PCR	43	IGHV4-AGR*02_S4099	IGHJ5-1*01	9	20	CSSRAKIFYSIGHDSGGRIDVW	DFPH-a
2-5	DFPH	39	2.262	Multiplex PCR	43	IGHV4-AGR*02_S4099	IGHJ5-1*01	9.1	20	CSTRANIYTDVGYSSGGRIDVW	DFPH-a
2-7	DFPH	39	0.288	Multiplex PCR	43	IGHV4-AGR*02_S4099	IGHJ5-1*01	9.4	20	CTSRAKIYSSGGSYSGGRIDVW	DFPH-a
2-8	DFPH	39	0.064	Multiplex PCR	43	IGHV4-AGR*02_S4099	IGHJ5-1*01	9.1	20	CSTRAKIYPGVGFSSVGRIDVW	DFPH-a
3-2	DFPH	39	0.078	Transcriptomics	43	IGHV4-AGR*02_S4099	IGHJ5-1*01	7.7	20	CTSRAKIYYGTDSYSGRRIDVW	DFPH-a
3-3	DFPH	39	0.085	Multiplex PCR	43	IGHV4-AGR*02_S4099	IGHJ5-1*01	8.7	20	CTTRAKVHSSVSYYYGGRIDVW	DFPH-a
5-2	DFPH	39	0.311	Multiplex PCR	43	IGHV4-AGR*02_S4099	IGHJ5-1*01	9.7	20	CTSRAKVHSSATFYYGGRIDVW	DFPH-a
5-4	DFPH	39	0.066	Multiplex PCR	43	IGHV4-AGR*02_S4099	IGHJ5-1*01	9.4	20	CTSRAKIFYSGGSSSGGRIDVW	DFPH-a
5-5	DFPH	39	0.022	Multiplex PCR	43	IGHV4-AGR*02_S4099	IGHJ5-1*01	8.4	20	CTSRAKIYYGTYSYAGGRIDVW	DFPH-a
5-6	DFPH	39	0.119	Multiplex PCR	43	IGHV4-AGR*02_S4099	IGHJ5-1*01	7.4	20	CTSRAKIFYSPSYDSGRRIDVW	DFPH-a
5-7	DFPH	39	5.343	Multiplex PCR	43	IGHV4-AGR*02_S4099	IGHJ5-1*01	9.7	20	CTSRAKVHSSVTQYYGGRIDVW	DFPH-a
6-2	DFPH	39	0.039	Multiplex PCR	43	IGHV4-AGR*02_S4099	IGHJ5-1*01	9	20	CTSRAKVHYSASYYYGGRIDVW	DFPH-a
6-5	DFPH	39	0.064	Multiplex PCR	43	IGHV4-AGR*02_S4099	IGHJ5-1*01	12	22	CSSRAKIYSGTTYSSASGGRIDAW	DFPH-a
6-6	DFPH	39	0.043	Multiplex PCR	43	IGHV4-AGR*02_S4099	IGHJ5-1*01	11.4	20	CTSRAEVYFSGGYYSGGRIDVW	DFPH-a
6-7	DFPH	39	0.034	Multiplex PCR	43	IGHV4-AGR*02_S4099	IGHJ5-1*01	7	20	CTSRAKIFYSVSSYSGRRIDVW	DFPH-a
6-8	DFPH	39	0.105	Multiplex PCR	43	IGHV4-AGR*02_S4099	IGHJ5-1*01	13	20	CSSRAKIYSGASFSPGGRIDVW	DFPH-a
7-2	DFPH	39	0.025	Transcriptomics	43	IGHV4-AGR*02_S4099	IGHJ5-1*01	7.7	20	CTSRAKIYSSGSYYAGGRIDVW	DFPH-a
7-5	DFPH	39	0.123	Multiplex PCR	43	IGHV4-AGR*02_S4099	IGHJ5-1*01	9	20	CATRAEIYYSSFYYSGGRIDVW	DFPH-a
7-6	DFPH	39	0.054	Multiplex PCR	43	IGHV4-AGR*02_S4099	IGHJ5-1*01	12	20	CTSRAKIYASGSYYSGGRIDVW	DFPH-a
7-7	DFPH	39	0.129	Multiplex PCR	43	IGHV4-AGR*02_S4099	IGHJ5-1*01	9.7	20	CTSRAKIYYSSTYYSGGRIDVW	DFPH-a
14-8	DFPH	39	0.053	Multiplex PCR	43	IGHV4-AGR*02_S4099	IGHJ5-1*01	9	20	CTSRAKVHSGATYYYGGRIDVW	DFPH-a
15-1	DFPH	39	0.182	Transcriptomics	43	IGHV4-AGR*02_S4099	IGHJ5-1*01	7.7	20	CVSRAKIYFSVGYDSGGRIDVW	DFPH-a
15-3	DFPH	39	0.022	Multiplex PCR	43	IGHV4-AGR*02_S4099	IGHJ5-1*01	9.8	20	CTARATVHSSASYYYGGRIDVW	DFPH-a
15-4	DFPH	39	0.113	Multiplex PCR	43	IGHV4-AGR*02_S4099	IGHJ5-1*01	9	20	CTSRAKVYSSASYYYGGRIDVW	DFPH-a
15-6	DFPH	39	0.022	Multiplex PCR	43	IGHV4-AGR*02_S4099	IGHJ5-1*01	9.4	22	CSSRAIIYYGTSSSSAVAGRIDVW	DFPH-a
15-8	DFPH	39	0.074	Multiplex PCR	43	IGHV4-AGR*02_S4099	IGHJ5-1*01	8.7	20	CTSRAKIYYSVSYYTGGRIDVW	DFPH-a
16-1	DFPH	39	0.185	Multiplex PCR	43	IGHV4-AGR*02_S4099	IGHJ5-1*01	8.7	20	CTSRAKVHSSASYYYGGRIDVW	DFPH-a
1-7	DFPH	39	0.072	Multiplex PCR	4	IGHV4-AGR*02_S4099	IGHJ4*01	10.8	6	CAAGIVTW	
2-1	DFPH	39	0.337	Multiplex PCR	4	IGHV4-AGR*02_S4099	IGHJ4*01	9.8	6	CVAGVVTW	
6-3	DFPH	39	1.644	Multiplex PCR	4	IGHV4-AGR*02_S4099	IGHJ4*01	10.7	6	CAGGIVTW	
7-1	DFPH	39	1.031	Multiplex PCR	4	IGHV4-AGR*02_S4099	IGHJ4*01	8.3	6	CAGGVVTW	
12-4	DFPH	18	0.252	Transcriptomics	2	IGHV4-AGR*02_S4099	IGHJ4*01	5	14	CARERLVSMTVDIDYW	DFPH-d
11-3	DFPH	20	0.229	Multiplex PCR	2	IGHV4-AGR*02_S4099	IGHJ4*01	6	14	CARERLVSMVVDTDYW	DFPH-d
11-4	DFPH	20	0.134	Multiplex PCR	1	IGHV4-AGR*02_S4099	IGHJ5-2*01	7.6	17	CAREAILHYCSGSACDAVW	
11-8	DFPH	20	127.800	Multiplex PCR	1	IGHV4-AGR*02_S4099	IGHJ4*01	7.3	15	CGRDGEANVEGATGDYW	
7-3	DFPH	39	0.192	Multiplex PCR	1	IGHV4-AGR*02_S4099	IGHJ4*01	5.6	17	CAREALRAYCSGSFCHDDW	
7-4	DFPH	39	0.043	Multiplex PCR	1	IGHV4-AFQ-U*01	IGHJ4*01	7.8	11	CAGSGTWNPHDYW	DFPH-b
15-5	DFPH	39	0.042	Multiplex PCR	1	IGHV3-AEH*01	IGHJ4*01	11.8	14	CANEGEDTGFSPLGSW	
15-7	DFPH	39	0.089	Multiplex PCR	1	IGHV1-AAU-U*01	IGHJ1*01	6.1	16	CARGPFGRINIHEYFEEW	
10-7	DFPH	20	0.320	N/A							
2-4	DFPH	39	0.079	N/A							

Table S1B

Table S1. Neutralization IC50s of single B cell culture supernatants and the corresponding antibody heavy chain sequence analysis. Related to Figures 1, 3, 6, and 7. A total of 87 supernatants from animal DFPH and other animals are shown. Heavy chain sequences were aligned on individualized IgDiscover database. The origin of the B cells, including animal name and time point, are shown. Supernatant neutralizing IC50s against BG505.N611Q virus are shown. Lineage ID was assigned to key lineages.

Table S1A. Supernatants from animal DFPH.

Table S1B. Supernatants from animals 13N008, A12V098, and A11V065.

Culture supernatant				Heavy chain sequencing and analysis							
ID	Animal	Week	BG505.N611Q IC50 (μg/mL)	Method	Clone size	VH	VJ	%SHM	CDR3 length	CDR3 sequence	Lineage ID
5-1	13N008	39	0.236	Multiplex PCR	3	IGHV3-AFR*01	IGHJ6*01	8.1	15	CARDNSYYFDSGYLDSW	
16-2	13N008	39	0.130	Transcriptomics	3	IGHV3-AFR*01	IGHJ6*01	4.1	15	CARDNSYYFDSGYLDSW	
16-4	13N008	39	0.244	Multiplex PCR	3	IGHV3-AFR*01	IGHJ6*01	6.1	15	CARDNSYYFDSGYLDSW	
7-8	13N008	39	0.355	Multiplex PCR	1	IGHV4-AFB-S*01	IGHJ4*01	11.7	14	CVREGESVGVIKGDYW	
4-3	A11V065	39	0.821	Multiplex PCR	6	IGHV4-ABB-S*01	IGHJ5-2*01	9.9	14	CMSPGTRFSSYSLEVW	A11V065-a
4-5	A11V065	39	0.787	Multiplex PCR	6	IGHV4-ABB-S*01	IGHJ5-2*01	13.7	14	CTSPGRGDSSYSLEFW	A11V065-a
4-6	A11V065	39	1.820	Multiplex PCR	6	IGHV4-ABB-S*01	IGHJ5-2*01	13.3	14	CTSPGRGDSSYSLEFW	A11V065-a
8-4	A11V065	39	2.046	Multiplex PCR	6	IGHV4-ABB-S*01	IGHJ5-2*01	11.7	14	CTSPGRGDSSYSLEFW	A11V065-a
14-5	A11V065	39	0.755	Multiplex PCR	6	IGHV4-ABB-S*01	IGHJ5-2*01	10.2	14	CMSPGTRFSSYSLEVW	A11V065-a
14-7	A11V065	39	0.385	Multiplex PCR	6	IGHV4-ABB-S*01	IGHJ5-2*01	10.9	14	CMSPGTRFSSYSLEVW	A11V065-a
3-8	A11V065	39	0.851	Transcriptomics	5	IGHV4-ADD*01	IGHJ5-2*01	11.9	14	CTSPGRGDSGYSMEFW	A11V065-b
4-2	A11V065	39	4.429	Multiplex PCR	5	IGHV4-ADD*01	IGHJ5-2*01	19.2	14	CTSPGRGDSSYSPEFW	A11V065-b
8-1	A11V065	39	0.432	Multiplex PCR	5	IGHV4-ADD*01	IGHJ5-2*01	13	14	CTSPGRGDSGYSMEFW	A11V065-b
8-6	A11V065	39	0.397	Transcriptomics	5	IGHV4-ADD*01	IGHJ5-2*01	12.6	14	CTSPGRGDSGYSMEFW	A11V065-b
14-3	A11V065	39	0.913	Transcriptomics	5	IGHV4-ADD*01	IGHJ5-2*01	12.6	14	CTSPGRGDSGYSMEFW	A11V065-b
10-2	A11V065	20	2.546	Multiplex PCR	4	IGHV4-ABB-S*01	IGHJ4*01	8.2	14	CATYDVDTVGTVVDYW	A11V065-c
10-3	A11V065	20	5.034	Multiplex PCR	4	IGHV4-ABB-S*01	IGHJ4*01	8.9	14	CATYDVDTVGTVVDYW	A11V065-c
10-4	A11V065	20	4.713	Multiplex PCR	4	IGHV4-ABB-S*01	IGHJ4*01	9.9	14	CATYDVDTVGTVVDYW	A11V065-c
4-4	A11V065	39	0.452	Multiplex PCR	4	IGHV4-ABB-S*01	IGHJ4*01	11.3	14	CATYDVDAIGTVVDYW	A11V065-c
4-7	A11V065	39	0.257	Multiplex PCR	2	IGHV3-AFR*01	IGHJ4*01	7.8	15	CGRDNSYYFDSGYLDFW	
8-3	A11V065	39	1.223	Multiplex PCR	2	IGHV3-AFR*01	IGHJ4*01	5.1	15	CARDRSYYFDGGYPDYW	
10-1	A11V065	20	1.042	Multiplex PCR	1	IGHV3-AET*03	IGHJ4*01	4.8	13	CSADYGNNYVYFDYW	
4-1	A11V065	39	1.187	Multiplex PCR	1	IGHV3-AEH*01	IGHJ5-2*01	21.4	14	CTSPGRGDSGYSMEFW	
8-2	A11V065	39	20.816	Multiplex PCR	1	IGHV4-AGR*01	IGHJ5-2*01	5.4	14	CTREPISSTYDSSDVW	
14-1	A11V065	39	0.333	Multiplex PCR	1	IGHV4-ABB-S*01	IGHJ4*01	12.6	6	CARAFDYW	
14-4	A11V065	39	0.063	Multiplex PCR	1	IGHV4-ABB-S*01_S3598	IGHJ1*01	8.8	12	CVTGDYYDDGPEIW	
14-6	A11V065	39	0.156	Multiplex PCR	1	IGHV4-ABB-S*01	IGHJ5-2*01	8.2	12	CASDPGGRTALDAW	
8-8	A11V065	39	0.077	N/A							
3-5	A12V098	39	0.130	Multiplex PCR	1	IGHV4-AGR*01	IGHJ5-1*01	13.6	6	CANGIVYW	
3-6	A12V098	39	0.800	Multiplex PCR	1	IGHV4-AGR*02_S4099	IGHJ4*01	7.3	14	CVAEPISTDGGADDSW	

Table S2

Table S2. Binding constants of reference mAbs and corresponding Fabs to BG505.DS.SOSIP.S613A trimer. Related to Figure 4. Binding constants of reference mAbs were measured using IgG-Trimer protocol. Binding constants of corresponding Fabs were measured using Trimer-Fab protocol. IgG binding was also measured in trimer ELISA. EC1.7 indicated the calculated concentration of IgG that reached OD450 of 1.7, which is usually about half-maximum binding. Endpoint titer indicated the lowest tested concentration of IgG that reached OD450 of 0.1, which is usually about twice the background binding.

mAb	IgG binding (IgG-Trimer method)						Fab binding (Trimer-Fab method)						Trimer ELISA	
	KD (M)	SE (KD)	ka (1/Ms)	SE (ka)	kd (1/s)	SE (kd)	KD (M)	SE (KD)	ka (1/Ms)	SE (ka)	kd (1/s)	SE (kd)	EC1.7 (μg/mL)	Endpoint titer (μg/mL)
112G11	2.26E-08	2.55E-10	7.53E+04	3.72E+02	1.70E-03	1.73E-05	3.36E-07	2.40E-09	1.04E+05	6.83E+02	3.49E-02	9.72E-05	0.0045	0.00064
106F6	2.30E-08	2.21E-10	5.50E+04	1.87E+02	1.27E-03	1.14E-05	1.43E-07	5.70E-10	5.43E+04	1.87E+02	7.75E-03	1.58E-05	0.0103	0.00064
113A12	3.66E-08	2.63E-10	4.66E+04	1.56E+02	1.71E-03	1.09E-05	7.71E-08	2.31E-10	6.67E+04	1.65E+02	5.15E-03	8.74E-06	0.0311	0.0032
106A4	1.52E-08	2.10E-10	6.59E+04	2.56E+02	9.99E-04	1.33E-05	5.57E-08	1.03E-10	7.49E+04	1.26E+02	4.17E-03	3.31E-06	0.0076	0.00064
113C9	3.26E-08	4.38E-10	3.42E+04	1.67E+02	1.11E-03	1.39E-05	4.78E-08	1.82E-10	4.82E+04	1.53E+02	2.30E-03	4.89E-06	0.1378	0.016
111A2	1.09E-08	1.69E-10	1.36E+05	9.12E+02	1.49E-03	2.08E-05	3.87E-08	7.12E-11	1.64E+05	2.75E+02	6.35E-03	4.72E-06	0.0090	0.00064
115B7	7.66E-09	2.68E-10	1.25E+05	1.27E+03	9.54E-04	3.19E-05	1.37E-08	2.16E-11	2.99E+05	4.24E+02	4.11E-03	2.85E-06	0.0050	0.00064
106E11	4.77E-09	2.24E-11	6.83E+04	1.93E+02	3.26E-04	1.22E-06	1.24E-08	6.74E-11	8.78E+04	2.21E+02	1.09E-03	5.25E-06	0.0078	0.00064
110H9	1.80E-09	2.21E-11	1.95E+04	3.71E+01	3.50E-05	4.25E-07	1.23E-08	2.25E-11	1.50E+04	1.88E+01	1.86E-04	2.46E-07	0.0096	0.00064
106D3	7.34E-09	4.44E-11	4.33E+04	1.67E+02	3.18E-04	1.48E-06	1.20E-08	1.76E-11	6.34E+04	7.53E+01	7.60E-04	6.49E-07	0.0189	0.0032
106C5	2.30E-09	1.95E-11	9.44E+04	3.70E+02	2.17E-04	1.63E-06	8.17E-09	3.81E-11	2.70E+05	7.51E+02	2.20E-03	8.25E-06	0.0044	0.00064
106C8	6.04E-09	2.77E-11	6.02E+04	1.79E+02	3.64E-04	1.27E-06	5.05E-09	8.02E-12	9.05E+04	9.23E+01	4.57E-04	5.56E-07	0.0078	0.00064
110H3	1.58E-09	1.83E-11	1.04E+05	4.48E+02	1.63E-04	1.75E-06	3.39E-09	5.02E-12	4.59E+04	4.74E+01	1.56E-04	1.65E-07	0.0043	0.00064
110H8	9.72E-10	1.20E-11	4.81E+04	7.55E+01	4.68E-05	5.74E-07	2.82E-09	5.18E-12	4.75E+04	5.29E+01	1.34E-04	1.96E-07	0.0063	0.00064
106F4	1.91E-09	1.67E-11	5.16E+04	1.13E+02	9.85E-05	8.34E-07	1.25E-09	3.33E-12	1.05E+05	2.01E+02	1.32E-04	2.42E-07	0.0037	0.00064
110E1	3.20E-10	1.25E-11	6.25E+04	1.25E+02	2.00E-05	7.77E-07	1.09E-09	2.87E-12	6.05E+04	6.51E+01	6.56E-05	1.59E-07	0.0035	0.00064
111A8	3.94E-10	1.73E-11	1.02E+05	4.59E+02	4.01E-05	1.76E-06	1.08E-09	1.30E-12	2.16E+05	2.01E+02	2.33E-04	1.78E-07	0.0030	0.000128
110F3	3.62E-10	1.34E-11	6.87E+04	1.61E+02	2.49E-05	9.20E-07	4.50E-10	1.08E-12	8.98E+04	1.05E+02	4.04E-05	<1.0E-07	0.0031	0.000128
106D1	4.06E-09	1.44E-10	4.56E+03	5.66E+01	1.85E-05	6.15E-07	3.05E-10	1.60E-11	8.44E+03	2.06E+01	2.58E-06	1.35E-07	0.0678	0.0032
111B6	7.04E-10	1.69E-11	6.59E+04	1.85E+02	4.64E-05	1.11E-06	2.17E-10	<1.0E-12	8.68E+04	8.22E+01	1.89E-05	<1.0E-07	0.0166	0.0032
111C3	4.59E-10	1.86E-11	7.48E+04	2.60E+02	3.43E-05	1.38E-06	2.07E-10	<1.0E-12	2.12E+05	1.32E+02	4.38E-05	<1.0E-07	0.0020	0.000128
107H11	1.74E-10	1.82E-11	2.43E+04	4.22E+01	4.21E-06	4.42E-07	1.29E-10	2.47E-12	2.95E+04	3.06E+01	3.79E-06	<1.0E-07	0.0062	0.00064

Table S3

Table S3. Binding constants of supernatants and corresponding Fabs to BG505.DS.SOSIP.S613A trimer. Related to Figure 5. Binding constants of supernatant IgGs were measured using IgG-Trimer protocol. Binding constants of corresponding Fabs were measured using Trimer-Fab protocol.

ID	Supernatant IgG binding (IgG-Trimer method)						Fab binding (Trimer-Fab method)					
	KD (M)	SE (KD)	ka (1/Ms)	SE (ka)	kd (1/s)	SE (kd)	KD (M)	SE (KD)	ka (1/Ms)	SE (ka)	kd (1/s)	SE (kd)
6-6	7.26E-09	2.38E-10	1.18E+05	1.01E+03	8.58E-04	2.72E-05	1.70E-08	7.49E-11	1.79E+05	7.26E+02	3.04E-03	5.37E-06
4-5	6.32E-09	1.62E-10	4.71E+03	6.00E+01	2.98E-05	6.61E-07	6.13E-09	1.33E-11	8.08E+03	5.10E+00	4.95E-05	1.03E-07
7-3	4.81E-09	1.41E-11	3.76E+04	5.10E+01	1.81E-04	4.69E-07	8.53E-09	2.30E-11	5.36E+04	9.51E+01	4.57E-04	9.28E-07
7-4	3.57E-09	2.68E-11	8.75E+04	3.85E+02	3.12E-04	1.90E-06	3.14E-09	6.18E-12	2.27E+05	3.27E+02	7.12E-04	9.54E-07
14-1	2.33E-09	4.69E-11	1.28E+04	4.79E+01	2.99E-05	5.89E-07	2.17E-08	1.13E-10	1.09E+04	4.59E+01	2.36E-04	7.29E-07
3-6	8.64E-10	3.29E-11	1.48E+04	4.05E+01	1.28E-05	4.87E-07	7.68E-09	1.95E-11	8.00E+03	8.92E+00	6.14E-05	1.40E-07
15-3	6.06E-10	1.88E-11	9.76E+04	4.48E+02	5.91E-05	1.82E-06	1.04E-10	1.70E-12	7.65E+04	4.35E+01	7.98E-06	1.30E-07
1-7	5.71E-10	1.47E-11	7.63E+04	2.14E+02	4.36E-05	1.12E-06	3.11E-10	1.86E-12	1.60E+05	1.09E+02	4.98E-05	2.95E-07
7-2	5.19E-10	1.67E-11	7.13E+04	2.14E+02	3.70E-05	1.19E-06	7.74E-10	1.93E-12	1.41E+05	1.05E+02	1.09E-04	2.59E-07
3-3	4.96E-10	7.95E-12	5.00E+04	1.51E+02	2.48E-05	3.90E-07	1.10E-10	1.08E-12	8.57E+04	3.83E+01	9.46E-06	<1.0E-07
6-7	3.49E-10	2.51E-11	3.56E+04	1.36E+02	1.24E-05	8.92E-07	2.91E-10	<1.0E-12	1.18E+05	6.13E+01	3.44E-05	<1.0E-07
5-6	1.54E-10	6.50E-12	3.29E+04	5.52E+01	5.05E-06	2.13E-07	1.64E-10	2.00E-12	1.10E+05	7.42E+01	1.81E-05	2.20E-07
2-3	1.12E-10	1.81E-11	5.31E+04	1.38E+02	5.95E-06	9.62E-07	2.47E-10	1.74E-12	5.05E+04	2.57E+01	1.25E-05	<1.0E-07

Table S4

Table S4. Neutralization activities of mAbs. Related to Figure 7. 10 culture wells with lowest supernatant IgG neutralization IC50s were selected for mAb recovery. 9 mAbs were expressed and tested for BG505 neutralization. 6 out of 9 mAbs neutralized wild-type BG505 with IC50 below 10 μg/mL and were further tested on a multi-clade panel of 9 wild-type HIV-1 strains as well as a control strain SIVmac251.30. Neutralization IC50s are shown with different background colors. Grey color indicates that either mAb was not recovered or neutralization was not tested.

Culture supernatant				mAb neutralization IC50 (μg/mL)											
ID	Animal	Week	BG505.N611Q IC50 (μg/mL)	BG505. N611Q	BG505 (clade A)	BB201.B42 (clade A)	BI369.9A (clade A)	246-F3.C10.2 (clade AC)	Q168.a2 (clade AD)	3988.25 (clade B)	BL01.DG (clade B)	CH117.4 (clade BC)	286.36 (clade C)	CNE58 (clade C)	SIVmac 251.30
15-3	DFPH	39	0.02	0.02	1.90	0.49	0.46	>50	6.44	1.36	1.33	1.36	13.64	>50	>50
15-6	DFPH	39	0.02	0.02	0.99	1.17	0.65	3.99	8.38	2.05	1.74	2.05	4.34	4.26	>50
5-5	DFPH	39	0.02	0.04	2.02	1.81	0.52	6.54	7.18	4.51	1.08	0.59	7.91	8.93	>50
7-2	DFPH	39	0.03	0.04	4.87	12.67	6.92	>50	25.60	>50	7.20	>50	>50	17.58	>50
11-1	DFPH	20	0.03	0.06	>50										
6-7	DFPH	39	0.03	0.04	1.48	1.32	0.80	4.78	5.72	1.96	1.90	0.73	3.33	2.84	>50
6-2	DFPH	39	0.04												
15-5	DFPH	39	0.04	0.13	10.81										
6-6	DFPH	39	0.04	0.05	5.51	>50	>50	>50	22.83	>50	33.31	27.01	19.23	>50	>50
7-4	DFPH	39	0.04	0.07	15.14										
mAb DFPH-a.01					1.96	1.99	1.49	5.93	12.72	4.42	3.18	1.53	4.24	3.34	>50

Legend
>50
10-50
1-10
0.1-1
0.01-1
